# Influence of Sulfur Source on Growth of In-Air Sprayed Ultrathin Film Sb_2_S_3_ for Enhanced Solar Cell Performance

**DOI:** 10.1021/acsami.5c17869

**Published:** 2025-11-11

**Authors:** Ernest A. Asare, Atanas Katerski, Merike Kriisa, Raavo Josepson, Victoria Rotaru, Maxim Guc, David Payno Zarceño, Alejandro Navarro-Güell, Raitis Grzibovskis, Aivars Vembris, Alejandro Pérez-Rodríguez, Edgardo Saucedo, Nicolae Spalatu, Malle Krunks, Ilona Oja Acik

**Affiliations:** † Department of Materials and Environmental Technology, 54561 Tallinn University of Technology, Ehitajate tee 5, 19086 Tallinn, Estonia; ‡ Division of Physics, Tallinn University of Technology, Ehitajate tee 5, 19086 Tallinn, Estonia; § 235241 Catalonia Institute for Energy Research (IREC), Jardins de les Dones de Negre 1, Sant Adrià de Besòs, 08930 Barcelona, Spain; ∥ Facultat de Fisica, Universitat de Barcelona (UB), C. Marti i Franques 1-11, 08028 Barcelona, Spain; ⊥ Departament d’Enginyeria Electronica i Biomedica, IN2UB, Universitat de Barcelona, C. Marti i Franques 1-11, 08028 Barcelona, Spain; # Institute of Solid State Physics, 61769 University of Latvia, Kengaraga Str. 8, Riga LV-1063, Latvia; ∇ Electronic Engineering Department, 16767 Universitat Politècnica de Catalunya (UPC), Av. d’Eduard Maristany 16, 08019 Barcelona, Spain; ○ Barcelona Center for Multiscale Science and Engineering, Universitat Politècnica de Catalunya (UPC), Av. d’Eduard Maristany 16, 08019 Barcelona, Spain

**Keywords:** antimony sulfide, solution method, precursor molar ratio, ultrathin films, solar cells

## Abstract

Addressing the critical need for scalable and efficient production of high-quality antimony sulfide (Sb_2_S_3_) ultrathin films for next-generation solar cell technologies, this study introduces a novel, industrially scalable approach utilizing ultrasonic spray pyrolysis with antimony trichloride (SbCl_3_) and thiourea precursors. This contrasts sharply with record Sb_2_S_3_ solar cells, often exceeding 200 nm and fabricated by using time-consuming chemical bath deposition, which presents challenges for tandem device integration. This work successfully fabricated high-quality 70 nm Sb_2_S_3_ ultrathin films. Extensive characterization revealed that excess thiourea addition in the Sb:S 1:6 ratio significantly reduced the growth rate, crucial for achieving ultrathin films, while simultaneously improving stoichiometry, morphology, and carrier transport, resulting in more homogeneous films. Devices fabricated with these optimized films demonstrated a substantial fill factor improvement, reaching 62%, comparable to the best reported values for Sb_2_S_3_ solar cells. This translated to a maximum power conversion efficiency of 5.3%. The films fabricated with the optimized thiourea addition in the Sb:S 1:6 ratio exhibited near-optimal stoichiometry, leading to a wider depletion width and improved device performance. This study proves the significance of precise precursor molar ratio control for high-quality ultrathin films, setting the stage for scalable Sb_2_S_3_ solar cells and advancing solution-processed photovoltaic technologies.

## Graphical abstract

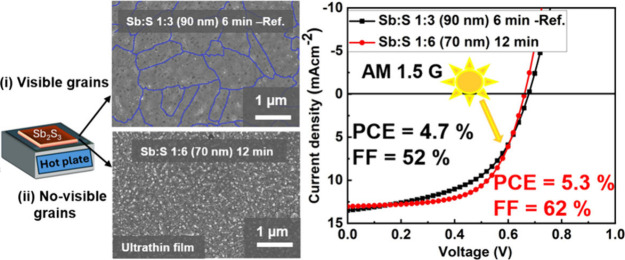

## Introduction

The relentless pursuit of sustainable and efficient photovoltaic (PV) technologies has ushered in a new era for low-cost earth-abundant materials. Antimony sulfide (Sb_2_S_3_) has emerged as a captivating contender in this arena, boasting exceptional stability, environmental friendliness, and a readily tunable wide band gap (1.7–1.8 eV) for efficient light absorption across a broad spectrum.^1,2^ With a Shockley–Queisser power conversion efficiency (PCE) limit of 28.64%, Sb_2_S_3_ shows promise as a top cell in Si tandem solar cells and other industrially established thin-film (TF) technologies like CdTe and CIGS.^3,4^ The thin-film architecture of Sb_2_S_3_ solar cells provides a significant weight reduction compared to other solar cell types thanks to the possibility of using lightweight and flexible substrates. This makes them well-suited for applications that require both portability and high performance, such as wearable devices, IoT technology, and space exploration.^5–7^ However, achieving the full potential of Sb_2_S_3_ solar cells requires the controlled synthesis of high-quality and uniform thin films with well-defined morphologies, crystal structures, and optimal electrical properties.^8,9^ Optimizing the material processing techniques is essential to improve the performance of Sb_2_S_3_ solar cells. Several research works have been devoted to creating novel methods of film deposition for this absorber material.^10,11^ Solution-based techniques like ultrasonic spray pyrolysis (USP) offer a compelling alternative to traditional methods like physical vapor deposition (PVD) and chemical vapor deposition (CVD) for Sb_2_S_3_ thin-film fabrication.^12,13^ The record-breaking efficiencies of Sb_2_S_3_ solar cells, 7.5% by Choi et. al in 2014 and the newest 8% by Wang et al., all featured the chemical bath deposition (CBD) method with an absorber thickness of around 200–1000 nm.^14,15^ CBD offers advantages such as low cost, simplicity, and high production capacity, but scaling up for large-scale production is not feasible and has limited substrate compatibility. USP boasts inherent advantages such as cost-effectiveness, scalability, and the ability to deposit uniform and adherent thin films on various substrates.^16–18^

The major advances in Sb_2_S_3_ solar cells over the past few years have primarily focused on absorber material optimization strategies such as doping,^19,20^ varying the Sb:S precursor molar ratio in solution,^21,22^ and interface modification.^15^ By varying the Sb:S molar ratio in their Sb_2_S_3_ precursors SbCl_3_:TU solutions, respectively, Seok et al. demonstrated the impact of composition engineering on device performance. Increasing the Sb:S precursor molar ratio in solution from 1:1.4 to 1:1.8 deposited via spin coating led to a linear increase in PCE (starting from 3.8%), reaching a maximum of 4.4%. However, further increasing the ratio to 1:2.2 resulted in a decrease in PCE to 3.9%.^22^ Wang et al. combined thiourea, thioacetamide, and sodium thiosulfate with antimony potassium tartrate, employing a multisulfur source strategy to adjust the grain size, surface morphology, and suppress impurity phases in Sb_2_S_3_ films, using CBD technology.^15^ This tactic successfully decreased the amount of vacancy-induced trapping centers, which enhanced device performance from a PCE of 5.4% to 8%. Choi et al. demonstrated that surface sulfurization using thioacetamide (TA) significantly enhanced the PCE of CBD-deposited Sb_2_S_3_ films by 7.5%.^14^ Regarding doping strategies, Jin et al.^19^ incorporated Cd into Sb_2_S_3_ via a hydrothermal process to promote (*hk*1)-oriented growth and achieve a 20% efficiency improvement (6.4%). Similarly, Liu et al.^20^ introduced Ce^3+^, forming a Ce_2_S_3_ interlayer that reduced the grain boundary density (from 1068 ± 40 to 327 ± 23 nm μm^–2^) and yielded a 7.66% PCE.

Notably, the precursor solution composition plays a pivotal role in dictating the final properties of the deposited Sb_2_S_3_ film.^23^ From material studies in our previous research, we have demonstrated the significant role of the precursor molar ratio during the spray pyrolysis deposition of various materials like SnS, CuInS_2_, and In_2_S_3_.^24–26^ These studies highlighted that an excess of thiourea (TU) in solution can provide enough liquid phase for metal sulfide formation, inhibit unwanted oxide phase formation, and impact film morphology behavior. Our initial investigation into the effect of SbCl_3_:TU molar ratios in the precursor solution for Sb_2_S_3_ film deposition revealed that a 1:2 ratio led to mixed Sb_2_S_3_/Sb_2_O_3_ phases, a 1:3 ratio produced partially crystalline Sb_2_S_3_ with reduced oxide content, and ratios ≥1:6 yielded phase-pure, oxide-free Sb_2_S_3_, indicating that excess thiourea effectively suppresses Sb_2_O_3_ formation.^24,25^ In another study, our group previously demonstrated the superior performance of Sb_2_S_3_ solar cells fabricated at 200 °C with a TU:SbEX (antimony ethyl xanthate) molar ratio of 4.5 (we explored TU:SbEX ratios from 0 to 4.5), achieving a record efficiency of 4.1% due to reduced oxidation, optimized band alignment, and favorable film morphology.^27^ In another study, Eensalu et al.^28^ established a platform and baseline using a 1:3 SbCl_3_ (Sb source):TU (S source) precursor molar ratio, yielding a record PCE of 4.7%. However, the effect of excess TU in SbCl_3_-TU precursor spray solutions (beyond that required for SbCl_3_-TU complex formation) on the formation of the Sb_2_S_3_ conformal thin film and solar cell properties remains unexplored. We investigated the impact of Sb_2_S_3_ film thickness on solar cell performance for the baseline Sb:S 1:3 precursor molar ratio, utilizing the same SbCl_3_ and TU precursors via ultrasonic spray pyrolysis as in this work. This prior work consistently revealed a trend of film and device degradation with increasing deposition time and thickness (or deposition cycles). Specifically for the Sb:S 1:3 ratio, the 70 nm film achieved a PCE of 4.4% with a fill factor of 55%. While the PCE showed a slight increase to 4.7% for the 100 nm film, performance drastically declined to 2.0% as the film thickness further increased to 150 nm. Although the influence of Sb_2_S_3_ layer thickness on solar cell performance using an Sb:S 1:3 precursor molar ratio has been previously investigated, a systematic study of this effect for films derived from 1:6 solutions is currently lacking in the literature. It is known that higher Sb:S molar ratios, specifically 1:6 and 1:9, enable the formation of oxide-free Sb_2_S_3_ layers via spray deposition in ambient air.^24–26^ Given that Sb_2_S_3_ hybrid solar cell output parameters are strongly dependent on the absorber layer thickness^28,29^ and considering the potential for higher purity in films obtained from 1:6 solutions, investigating the effect of film thickness (controlled via spray cycles) on solar cell output characteristics using the Sb:S 1:6 molar ratio is of significant importance.

This study investigates the impact of excess TU in SbCl_3_-TU precursors on Sb_2_S_3_ thin-film properties by exploring the impact of the Sb:S 1:6 precursor molar ratio and comparing it to the established 1:3 ratio. Understanding how the ratio affects properties like crystallinity, morphology, stoichiometry, and charge carrier properties is crucial for optimizing the performance of the Sb_2_S_3_ solar cell. Furthermore, the synthesis of high-quality Sb_2_S_3_ thin films for solar cell applications with an industrial-scale deposition technique still remains a challenge.

By optimizing the Sb:S 1:6 molar ratio in SbCl_3_-TU precursors and its fabrication process, we posit that this will enhance the quality of Sb_2_S_3_ films and improve their optoelectronic properties, ultimately boosting the efficiency and reproducibility of Sb_2_S_3_-based solar cells while demonstrating the robustness of the less time-consuming and cost-effective industrial-scale USP deposition technique. This work presents significant advancements in Sb_2_S_3_ solar cell research in several key aspects. First, we report an optimized fabrication protocol to obtain high-quality ultrathin Sb_2_S_3_ films (70 nm) with an enhanced fill factor (FF) of 62% and PCE of 5.3% measured under standard AM 1.5G test conditions. This innovation hinges on the unique 1:6 Sb:S molar ratio employed in the SbCl_3_-TU precursor solution, paving the way for a more streamlined and reliable fabrication process. Additionally, the synergistic combination of high performance, optimized precursor ratio, and scalable fabrication offers immense potential for advancing Sb_2_S_3_ solar cell technology, and this work provides valuable insights for industry and future research.

## Results and Discussion

### Effect of Precursor Molar Ratio on USP-Deposited Sb_2_S_3_ Thin-Film Properties

An ultrasonic spray deposition method is employed to deposit Sb_2_S_3_ photoactive films on glass/FTO/TiO_2_ substrates, where SbCl_3_ and TU precursors are used as Sb and S sources, respectively, as shown in Figure 1a. The Sb:S 1:3 precursor molar ratio in solution is prepared for the reference, deposited at 40 cycles at a substrate temperature of 185 °C and annealed at 270 °C in N_2_ for 6 min, repeating the same conditions as those of the record cell reported by our group with the USP deposition method.^28^ A different precursor molar ratio, Sb:S 1:6 in solution, is prepared and deposited at different deposition cycles, 40, 50, 60, and 70 cycles, at the same substrate temperature 185 °C on different parallel substrates and annealed at 270 °C in N_2_ for 6 min. It must be well noted that all experimental conditions were kept constant except the number of deposition cycles, which was varied for the Sb:S 1:6 precursor molar ratio in solution.

**Figure 1:**
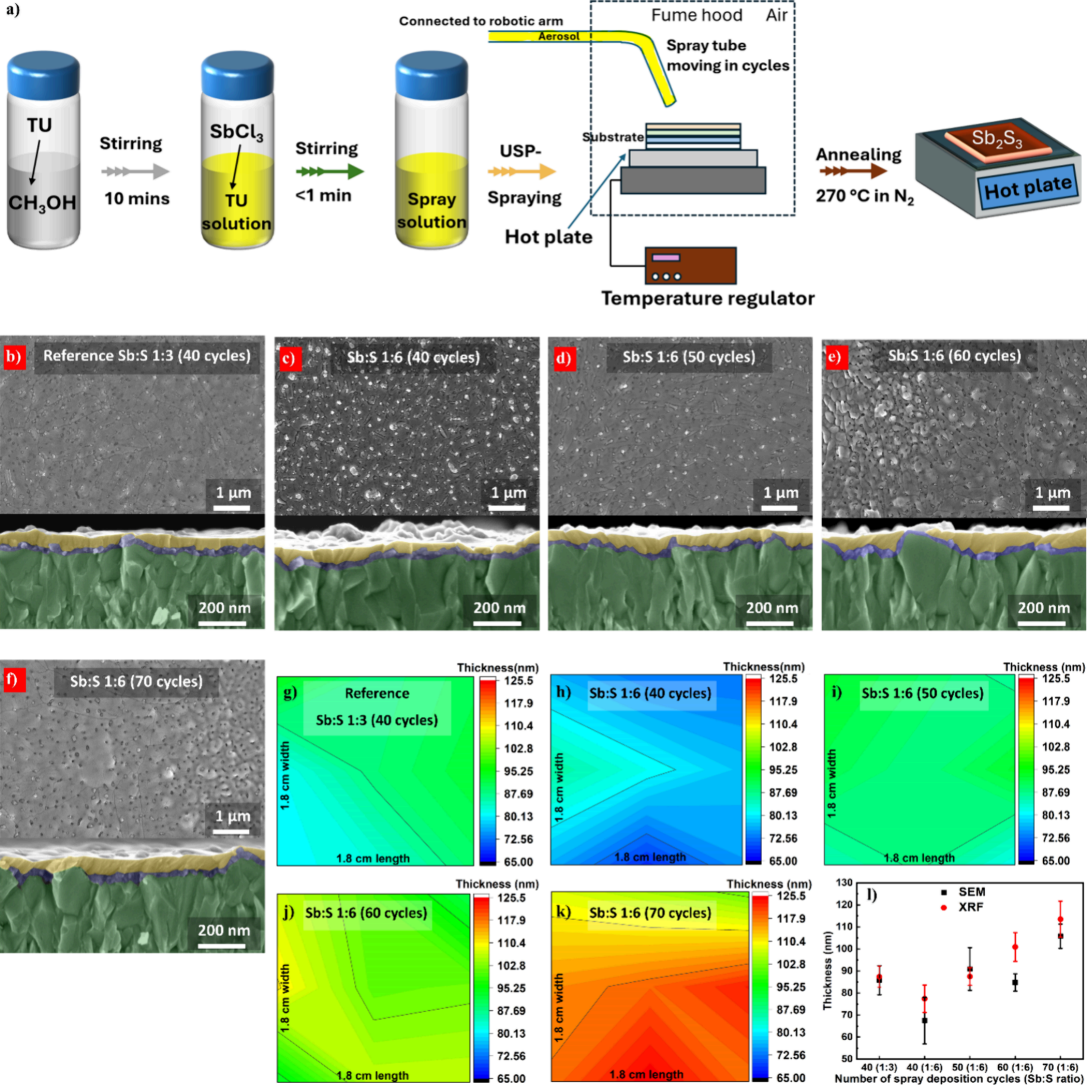
(a) Schematic illustration of the solution-processing of Sb_2_S_3_ thin films using an ultrasonic spray method. Surface and cross-sectional SEM images of Sb_2_S_3_ films (annealed at 270 °C for 6 min) grown on FTO (in green)/TiO_2_ (in blue) substrates using varying precursor molar ratios in solution and deposition cycles to achieve different thicknesses: (b) reference Sb:S 1:3 (40 cycles), (c) Sb:S 1:6 (40 cycles), (d) Sb:S 1:6 (50 cycles), (e) Sb:S 1:6 (60 cycles), and (f) Sb:S 1:6 (70 cycles). XRF thickness measurements for these films: (g) reference Sb:S 1:3 (40 cycles), (h) Sb:S 1:6 (40 cycles), (i) Sb:S 1:6 (50 cycles), (j) Sb:S 1:6 (60 cycles), and (k) Sb:S 1:6 (70 cycles). (l) Comparison of Sb_2_S_3_ film thickness measured by SEM and XRF for different Sb:S precursor molar ratios and deposition cycles.

Surface and cross-sectional scanning electron microscopy (SEM) characterizations were conducted to thoroughly understand the Sb_2_S_3_ film growth on glass/FTO/TiO_2_ substrates. Figure 1b presents both the surface and cross-sectional SEM images of the annealed reference Sb:S 1:3 (40 cycles) film. The white spots observed on the film surface are identified as pinholes, resulting from incomplete Sb_2_S_3_ film coverage that allows underlayer TiO_2_ to penetrate or remain exposed, likely due to the underlying FTO’s roughness. Although the presence of such pinholes might generate pathways for shunting and might have an implication on device performance, it has been shown by Kaienburg et al.^30^ that for contact between the ETL and HTL, which is known as non-ohmic shunting, the P3HT (HTL) mitigates such effects. For the Sb:S 1:6 (40 cycles) film shown in Figure 1c, these spots are more predominant. This suggests that the thinner Sb_2_S_3_ film allows for greater penetration and exposure of the underlying TiO_2_ layer. To investigate the impact of increased thickness, the deposition cycles for the Sb:S 1:6 molar ratio were varied from 50 to 70 cycles. Figure 1d, showing the 50 cycles film, reveals the onset of dome-like structures along the surface, likely caused by particle agglomeration. Despite this, there is an improved coverage of the TiO_2_ layer, resulting in fewer white spots and, thus, reduced TiO_2_ penetration across the Sb_2_S_3_ film. As the deposition cycles increased to 60 and 70 (Figure 1e,f), these dome-like structures became more pronounced. Such significant surface irregularities can impede the formation of a good electrical contact with the back metal conductor. Furthermore, cross-sectional SEM analysis (Figure 1b–f) for the Sb:S 1:6 (60 cycles) sample (Figure 1e) reveals that complete coverage of the underlying layers by the Sb_2_S_3_ film remains challenging even with a higher number of deposition cycles, which is likely attributable to the inherent roughness of the FTO substrate. Film thicknesses were quantitatively determined from SEM cross-sectional images by averaging the measurements at four different points. The reference Sb:S 1:3 (40 cycles) sample exhibited an average thickness of approximately 86 nm, while the Sb:S 1:6 (40 cycles) sample was approximately 68 nm thick. Increasing the deposition cycles for the Sb:S 1:6 films to 50, 60, and 70 resulted in approximate average thicknesses of 91, 85, and 106 nm, respectively.

To evaluate large-area film thickness uniformity, X-ray fluorescence (XRF) analysis was performed across a three-by-three matrix on the surface. A heat map was subsequently generated to visualize the spatial distribution of the measured thicknesses. The XRF image of the reference Sb:S 1:3 (40 cycles) shown in Figure 1g gives an average thickness of 90 nm, correlating well with the thickness determined from SEM, which are both close to literature values of USP-Sb_2_S_3_ film deposited in 40 cycles with an Sb:S 1:3 molar ratio.^31^ Figure 1h–k presents XRF images of Sb_2_S_3_ films grown with an Sb:S molar ratio of 1:6 and varying deposition cycles (40, 50, 60, and 70) to assess the uniformity of film thickness. These thicknesses show a close relation with the SEM determined thicknesses (Figure 1l), except for the Sb:S 1:6 (60 cycles) sample, where there is a minor deviation. This minor deviation is attributed to the localized nature of SEM cross-sectional analysis, which provides thickness information from a limited area.

Since the spatially resolved XRF data obtained from the mapping procedure allow for a robust evaluation of the film’s large-area uniformity, the thicknesses obtained from XRF results (Figure 1g–k) concluded that the approximate thickness of the reference Sb:S 1:3 (40 cycles) is 90 nm, while those of the Sb:S 1:6 (40 cycles), Sb:S 1:6 (50 cycles), Sb:S 1:6 (60 cycles), and Sb:S 1:6 (70 cycles) samples are 70, 90, 100, and 120 nm, respectively (as summarized in Table S1 in the Supporting Information). Hence in the subsequent discussion, the number of cycles is replaced with the approximated film thicknesses of the Sb_2_S_3_ films for more clarity. From the results, it can also be extracted that the excess of TU seems to reduce the growth rate of the Sb_2_S_3_ films, since 40 deposition cycles grew only 70 nm Sb_2_S_3_ films, whereas the same deposition cycles produced 90 nm Sb_2_S_3_ for the Sb:S 1:3 reference. We have consistently observed a slower growth rate of Sb_2_S_3_ films when spraying solutions with an Sb:S molar ratio of 1:6 compared to 1:3 at 200–220 °C.^32^ This slower growth is likely attributed to the increased concentration of thiourea (TU) in the 1:6 solution. In prior investigations into the synthesis of copper indium disulfide (CuInS_2_) films from aqueous solutions of copper chloride CuCl_2_, indium chloride (InCl_3_), and thiourea (SC­(NH_2_)_2_), we employed simultaneous thermogravimetry/differential thermal analysis/evolved gas analysis–mass spectrometry (TG/DTA/EGA-MS) to monitor precursor decomposition at Cu:In:S molar ratios of 1:1:3 and 1:1:6 (with excess thiourea).^24,33^ Our analysis revealed that the thermal degradation of the 1:1:3 precursor system, specifically within the temperature range 200–305 °C, resulted in a mass loss of 21.3%. In contrast, the inclusion of excess thiourea in the 1:1:6 molar ratio led to a significantly higher mass loss of 42.6%, representing approximately a 50% increase compared with the lower thiourea counterpart. This differential mass loss likely explains why identical spray cycles (40 cycles) for Sb:S 1:3 and 1:6 molar ratios, under otherwise identical deposition conditions, result in varying final thin-film thicknesses. This substantial volatilization was directly attributed to the presence of excess thiourea and was observed to impede the growth rate of the CuInS_2_ films. EGA-MS data identified the primary gaseous decomposition products as CS_2_, NH_3_, H_2_NCN, and HNCS, indicating the melting and subsequent decomposition of adjacent thiourea molecules. Further oxidation of these species yielded additional volatile byproducts, including COS, SO_2_, HCN, and CO_2_. Hence, the generation of these volatile byproducts (CS_2_, NH_3_, H_2_NCN, HNCS, COS, SO_2_, HCN, and CO_2_)^26,34–36^ at these temperatures can disrupt the formation of stable Sb_2_S_3_ nuclei on the active TiO_2_ substrate surface, likely due to increased bubbling and turbulence within the liquid phase reducing the Sb_2_S_3_ growth rate. This may contribute to the reason why spraying the same number of cycles (40 cycles) for Sb:S 1:3 and 1:6 molar ratios under the same deposition conditions yields different thin-film thickness. A similar trend of slower growth rates at higher thiourea concentrations has also been consistently observed in our previous studies involving In_2_S_3_ and SnS.^25,26^

X-ray diffraction (XRD) study (Figure 2a) was carried out to look into the effects of the 1:6 Sb:S molar ratio in solution on the structural properties of the annealed Sb_2_S_3_ films deposited on glass/FTO/TiO_2_ substrates with varying thicknesses from 70 to 120 nm. The acquired XRD patterns showed well-defined and sharp peaks consistent with the crystalline orthorhombic crystal structure of stibnite Sb_2_S_3_ (ICSD 01-075-4012) for all thicknesses, as shown in Figure 2a, with lattice constants *a* = 11.30 ± 0.08 Å, *b* = 11.23 ± 0.06 Å, and *c* = 3.82 ± 0.01 Å, comparable to literature values and *Pnma* space group symmetry.^19,37^ The average Scherrer crystallite size of the (*hk*1) dominant (011), (111), and (211) diffraction peaks as summarized in Table S3 (in the Supporting Information), determined at 24.64°, 25.08°, and 29.36°, respectively, show similar values.

**Figure 2:**
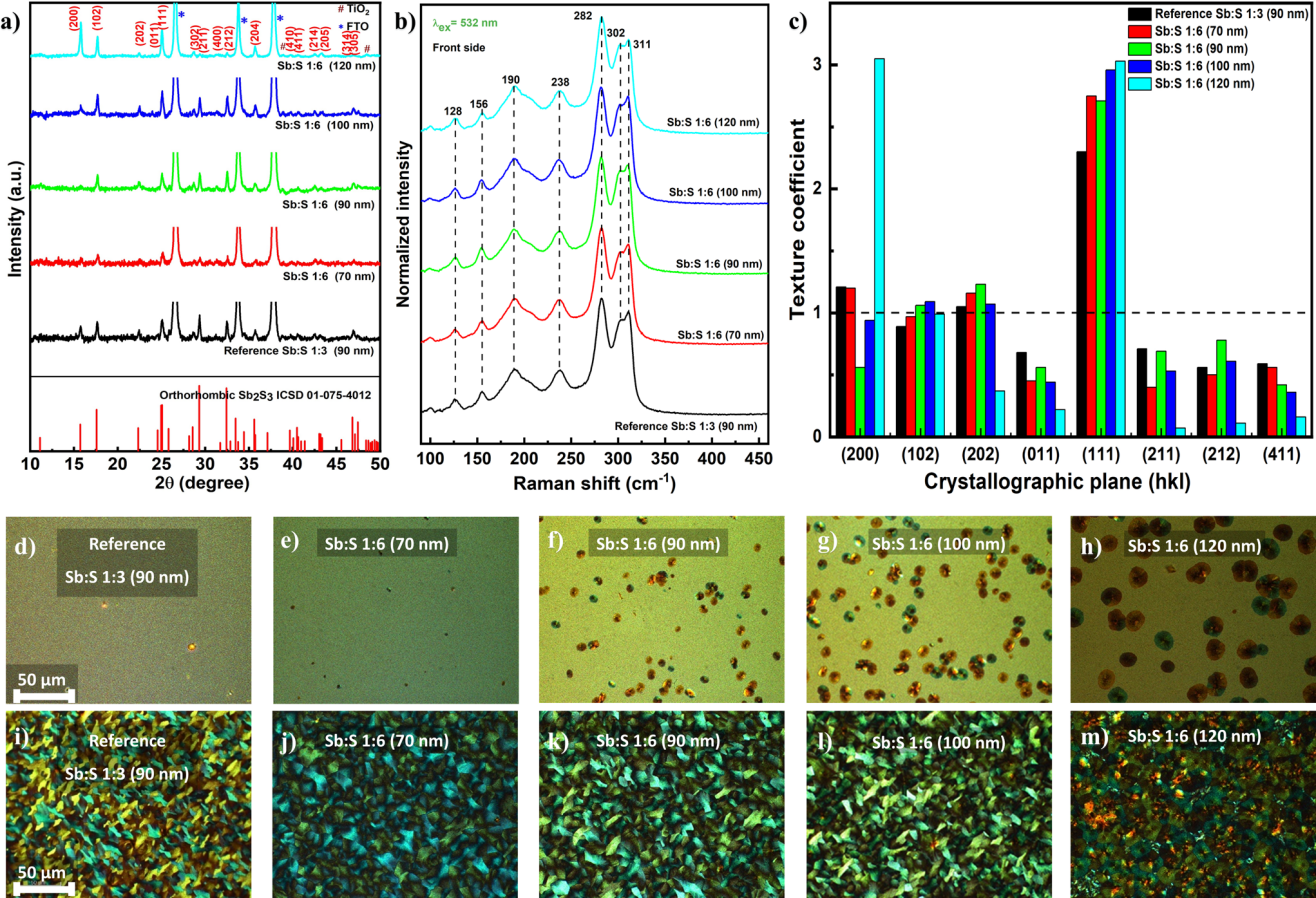
(a) XRD patterns, (b) Raman spectra, and (c) texture coefficients of Sb_2_S_3_ thin films with varying Sb:S precursor molar ratios and film thicknesses annealed for 6 min. Optical microscopy images of as-deposited Sb_2_S_3_ films in polarized light mode: (d) reference Sb:S 1:3 (90 nm), (e) Sb:S 1:6 (70 nm), (f) Sb:S 1:6 (90 nm), (g) Sb:S 1:6 (100 nm), and (h) Sb:S 1:6 (120 nm). Optical microscopy images of the same films after annealing for 6 min in polarized light mode: (i) reference Sb:S 1:3 (90 nm), (j) Sb:S 1:6 (70 nm), (k) Sb:S 1:6 (90 nm), (l) Sb:S 1:6 (100 nm), and (m) Sb:S 1:6 (120 nm).

In addition to XRD, Raman spectroscopy was used to further clarify the phase composition of the Sb:S 1:6 Sb_2_S_3_ films. Certain local atomic configurations that may not be seen by XRD can be found by Raman spectroscopy. The Raman spectra showed peaks at 128, 156, 190, 238, 282, 302, and 311 cm^–1^, which matched vibrational modes of crystalline Sb_2_S_3_ films that have been previously established in the literature.^38,39^ However, there are some variations in the relative intensity of some peaks. In the case of the 532 nm excitation wavelength, these variations are mainly seen in the 300–311 cm^–1^ spectral region that could be related to either changes in the crystallographic orientation or slight changes in the composition of the thin films and the presence of point defects (Figure 2b).^38^ According to previous studies,^38^ changes in crystallographic orientation should also result in changes in the relative intensity of the peaks around 190 cm^–1^, and these changes were clearly observed only for the Sb:S 1:6 120 nm sample, in accordance with the strong texture change as discussed below. This difference in relative intensity of the peaks around 190 cm^–1^ in the Sb:S 1:6 120 nm sample was also found in the spectra measured from the back side of the samples (Figure S1c), confirming the in-depth homogeneity of the sample. On the other hand, variations of composition should result in more clear variations in the low wavenumber peaks in the spectra measured under 632.8 nm excitation.^38^ These spectra were measured and analyzed (see Figure S1d,e); however, no strong variations were found in the low wavenumber peaks, confirming just slight variation in the composition between different samples. Finally, the full width at half-maximum (fwhm) of the main Raman peak at 282 cm^–1^ was analyzed (Figure S1f,g), and no significant difference of this value was found for all of the annealed thin films, denoting no strong variation in the crystalline quality of these samples. Importantly, neither Raman spectroscopy nor X-ray diffraction analyses detected the presence of a significant concentration of Sb_2_O_3_ or any other secondary phases. The precursor molar ratio of Sb:S 1:6 resulted in insignificant alterations in the structural and morphological properties of the Sb_2_S_3_ film compared to the Sb:S 1:3 reference sample.

The only major difference appeared (XRD patterns, Figure 2a) in the abrupt increase of the intensity of the peaks assigned to (*hk*0) planes, like the (200) plane situated at 2θ = 15.78° for the 120 nm Sb:S 1:6 thin film. This is probably due to partial-crystallization of the thicker Sb_2_S_3_ thin film, which starts to occur already during deposition, as confirmed by XRD patterns and Raman spectra for the as-deposited films (Figure S1a,b, Supporting Information). This partial-crystallization favors the horizontal growth of the (200) plane, as shown in the as-deposited XRD patterns (Figure S2a), and may make the sample less suitable for charge transport.^40^ The texture coefficient (TC) in Figure 2c was computed using the Harris formula in eq S1 (in the Supporting Information) in order to evaluate the preference for crystallographic orientation and ascertain the relative distribution of crystal growth directions in the Sb:S 1:6 Sb_2_S_3_ films.^41^

If the TC is higher or smaller than unity, it means that the film’s packing density and degree of preferential orientation along a specific crystal plane are higher or lower than they would be for a powdered material.^42,43^ For the reference and the entire Sb:S 1:6 films, the TC of only (111) among the (*hk*1) planes exceeded unity, thus indicating the preferred orientation of the absorber grains along the [111] direction. However, the (102) and (202) planes reached unity for all Sb_2_S_3_ films, suggesting pronounced orientation preference together with the (111) plane. Another important feature to note is the exceptional increase in the intensity of the peak of the (200) plane compared with the rest of the (*hk*1) planes for the 120 nm sample. The (200) plane exhibited a notable difference between the Sb:S 1:6 90 nm and the reference sample, with the latter showing a TC greater than unity. However, it can be noted that all grown Sb:S 1:6 Sb_2_S_3_ films and the reference 1:3 sample promoted vertical growth of the (Sb_4_S_6_) ribbons.

To further prove the claim of partial-crystallization during deposition of thicker films, optical microscopy images in polarized mode of as-deposited and annealed samples of the Sb:S 1:6 Sb_2_S_3_ were taken (Figure 2d–m). Optical microscopy images of as-deposited amorphous samples show the plane surface for the reference and Sb:S 1:6 (70 nm). Crystallization starts to occur, which is seen as dark brownish and dark green disc-like shapes, as we grow thicker films. These shapes increase and spread respectively from Sb:S 1:6 90 to 120 nm. This correlates well with the XRD patterns and Raman spectra (Figure S1a,b) measured in the as-deposited Sb_2_S_3_ thin films. During the postcrystallization with annealing for 6 min, the partially crystallized centers turn into dark spots that are seen here as dark yellowish spots in the annealed samples more predominant in Figure 2m, which can serve as defect centers and may affect the device performance afterward. The optical properties of the Sb_2_S_3_ thin films were characterized by using transmittance, reflectance, and absorption measurements, as shown in Figure S2a–c. A slight increase in absorption was observed with increasing film thickness, while thinner films (70 nm) exhibited higher transmittance and reflectance. The average visible transmittance (AVT) of the back-contact-less ITO/TiO_2_/Sb_2_S_3_ stack, calculated using eq S2 in the wavelength range of 380–740 nm, revealed that the ultrathin Sb:S 1:6 (70 nm) film had an AVT of 28%. This value decreased to 16% as the Sb_2_S_3_ thickness increased from 90 to 120 nm, as summarized in Table 1.

**Table 1:** Photovoltaic Performance Parameters of the Reference Sb:S 1:3 (90 nm) and Sb:S 1:6 Molar Ratio Samples with Varying Thicknesses Annealed for 6 min, Measured under AM 1.5G Illumination Conditions

Sb:S (thickness)	*V*_OC_ (mV)	*J*_SC_ (mA cm^–2^)	FF (%)	PCE (%)	*J*_0_ (mA cm^–2^)	*R*_S_ (Ω cm^2^)	*R*_Sh_ (Ω cm^2^)	AVT (%)
1:3 (90 nm) reference	676	13.4	52	4.7	3.54 × 10^–5^	1.5	345	19
	702 ± 12^a^	12.1 ± 0.6	51.8 ± 0.7	4.4 ± 0.2		1.4 ± 0.3	333 ± 11	
1:6 (70 nm)	602	12.3	58	4.3	5.88 × 10^–6^	1	1157	28
	631 ± 39	11.0 ± 1	58.8 ± 0.9	4.1 ± 0.1		0.9 ± 0.1	1207 ± 140	
1:6 (90 nm)	690	10.6	51	3.7	2.45 × 10^–5^	1	356	21
	662 ± 36	10.3 ± 0.6	52.4 ± 2.3	3.5 ± 0.3		1.1 ± 0.1	435 ± 202	
1:6 (100 nm)	654	10	59	3.8	4.10 × 10^–4^	1	964	19
	663 ± 30	10.1 ± 0.5	54.3 ± 4.2	3.6 ± 0.3		1.0 ± 0.2	611 ± 304	
1:6 (120 nm)	635	12.9	55	4.5	9.16 × 10^–5^	0.7	642	16
	662 ± 17	11.1 ± 1.0	54.4 ± 2.7	4.0 ± 0.4		1.1 ± 0.3	580 ± 188	

a Average and standard deviation.

### Effect of Precursor Molar Ratio on USP-Deposited Sb_2_S_3_ Device Performance

Having resolved the influence of the Sb:S 1:6 precursor molar ratio in solution on the morphology and structural properties of USP-Sb_2_S_3_ thin films with varying thicknesses, we will now examine the impact of these variations on PV performance. The devices were fabricated in a planar configuration (Figure 3a). The current density–voltage (*J*–*V*) characteristics of the most representative devices are presented in Figure 3b. Statistical plots are shown in Figure 3c–h, and the corresponding photoelectrical parameters, extracted from seven devices for each variation, are summarized in Table 1.

**Figure 3:**
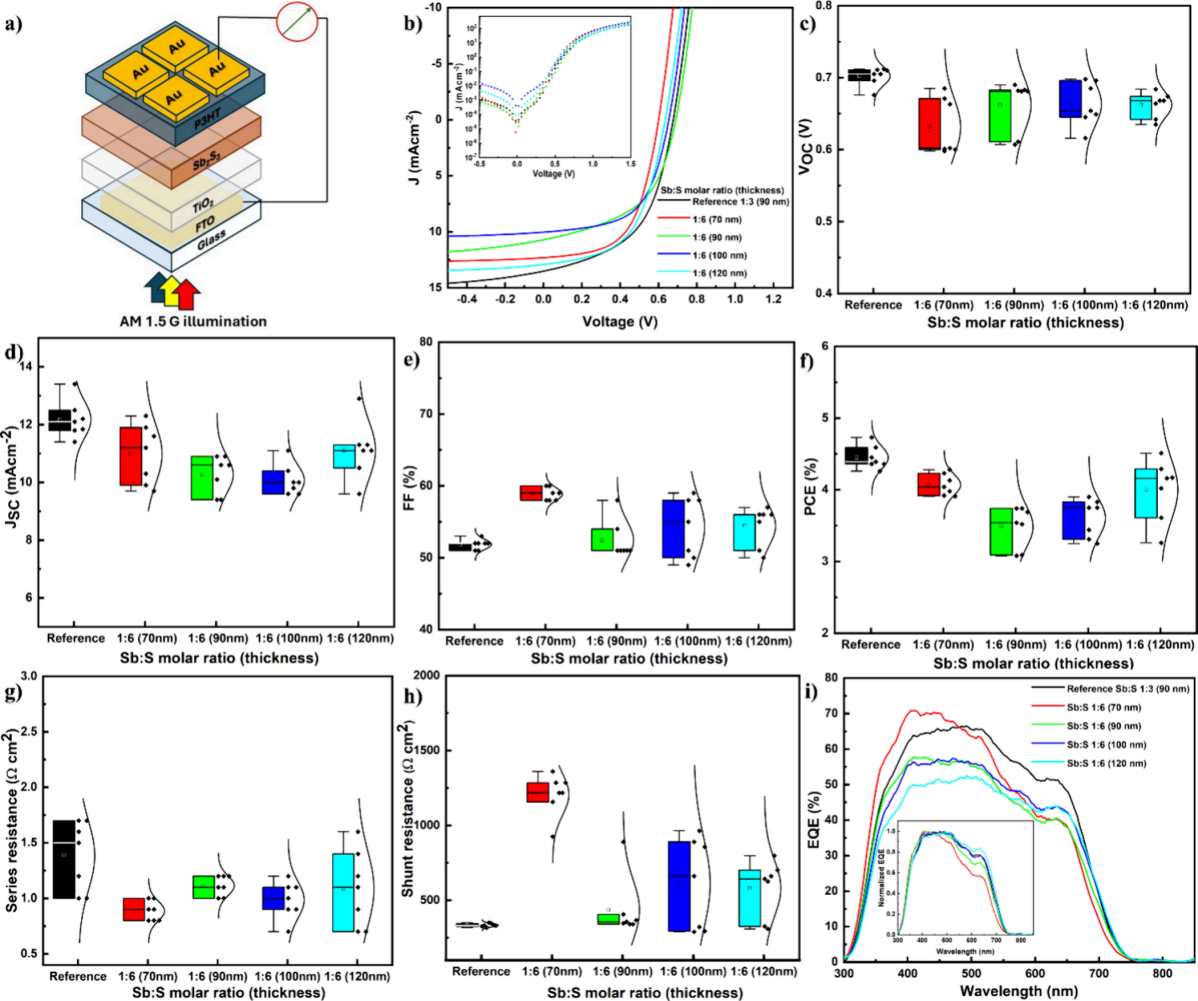
(a) Schematic Sb_2_S_3_ solar cell representation, (b) illuminated *J*–*V* curve of highest-performing devices (inset: dark *J*–*V* curve), and statistical analysis of (c) *V*_OC_, (d) *J*_SC_, (e) FF, (f) PCE, (g) series resistance, and (h) shunt resistance for solar cells measured under AM 1.5G illumination as a function of Sb:S precursor molar ratio with varying thicknesses. (i) External quantum efficiency (EQE) (inset: normalized EQE) of the best-performing cells based on the Sb:S precursor molar ratio and varying thickness. The reference sample is Sb:S 1:3 (90 nm).

As the thickness of the Sb:S 1:6 devices increased from 70 to 90, 100, and 120 nm, the open-circuit voltage *V*_OC_ remained relatively constant, ranging from 0.6 to 0.7 V. In comparison, the reference system (Sb:S 1:3 90 nm) exhibited *V*_OC_ values within the range of 0.676–0.705 V, showing no significant difference from the best Sb:S 1:6 devices. Now coming back to previously observed uncovered spots (Figure 1b–f), one could expect that the presence of such pinholes might generate pathways for shunting, impacting *V*_OC_ and overall performance. Such a phenomenon has already been reported by Kaienburg et al.,^30^ showing that while pinholes can lead to shunting, robust ETL/HTL interfaces can effectively block these pathways. Specifically, they found that TiO_2_/P3HT contacts effectively suppress non-ohmic shunting and prevent fill factor (FF) degradation.

However, the reverse saturation current *J*_0_ (calculated from the 1 diode model), an important parameter for the p–n junction quality, which can be extracted from the dark *J*–*V* curves shown in Figure 3b and is presented in Table 1, reveals slight changes. The *J*_0_ reveals comparable p–n junction quality across the reference Sb:S 1:3 (90 nm) and most of the Sb:S 1:6 films (90 and 120 nm), which may suggest a similar recombination mechanism. The Sb:S 1:6 (70 nm) exhibits a significantly lower *J*_0_ (order of 10^–6^ mA cm^–2^), indicating a superior junction with reduced leakage. Conversely, the Sb:S 1:6 (100 nm) film shows a substantially higher *J*_0_ (order of 10^–4^ mA cm^–2^), which may imply degraded junction characteristics. This suggests that the introduction of the Sb:S 1:6 precursor may not effectively suppress recombination losses within the solar cell, as *V*_OC_ and *J*_0_ are directly correlated with dominant recombination processes.^5^ No significant *J*_SC_ increase can be observed as the thickness is increased from 70 nm (12.3 mA cm^–2^) to 120 nm (12.9 mA cm^–2^). While one might expect *J*_SC_ to increase with thicker films until it reaches a saturation point, this is not always straightforward.^37^ *J*_SC_ saturates after approximately 40 min of deposition, and precrystallization begins to occur, favoring the horizontal growth of Sb_2_S_3_ ribbons as aforementioned. Dome-like structures at the surface (Figure 1e,f) may hinder charge transport and introduce parasitic absorption and defect centers, ultimately deteriorating cell performance. The thinner 70 nm Sb:S 1:6 device, fabricated with a deposition time of 40 min, exhibited a significantly improved shunt resistance (*R*_Sh_) of approximately 1.1 kΩ cm^2^, which is four times greater than that of the reference device, 0.3 kΩ cm^2^. This improvement led to an improved fill factor (FF) of 58% for the best device. However, as the deposition time exceeded 40 min and thicker films were grown, the morphology of the device worsened, and current leakage pathways increased, resulting in a decrease in *R*_Sh_ and consequently FF. Overall, all Sb:S 1:6 devices demonstrated improved FF compared to the reference cell, which may also be attributed to their lower series resistance (*R*_S_) ranging from 0.8 to 1.1 Ω cm^2^ for most devices. The 70 nm Sb:S 1:6 device achieved a PCE of 4.3%, slightly lower than the reference Sb:S 1:3 (90 nm) device’s PCE of 4.7% (90 nm thick), like previously reported results for USP-Sb_2_S_3_ devices with an Sb:S 1:3 molar ratio using a cell area of 7.02 mm^2^.

The external quantum efficiency (EQE) of the devices was characterized to assess their spectral response, as shown in Figure 3i. EQE quantifies the efficiency of converting incident photons into collected charge carriers at a specific wavelength, which is described by eq 1:^44^

1 $$\mathrm{EQE}=\frac{\mathrm{electron flux}}{\mathrm{incident photon flux}}=\frac{J_{\mathrm{ph}}\left(\lambda ,v\right)}{I\left(\lambda \right)}\frac{hv}{q}$$

where *J*_ph_ represents the photocurrent density, *I*(λ) denotes the incident light intensity at a specific wavelength, *q* is the elementary charge, and *hv* corresponds to the photon energy. EQE measurements reveal that the short-wavelength region (300–400 nm) is particularly sensitive to variations in the electron transport layer (ETL), specifically the USP-deposited TiO_2_. This sensitivity likely stems from the inherent nonuniformity of these ultrathin (∼30 nm) TiO_2_ films. For Sb:S 1:6 devices, we observe a notable decrease in EQE response within the blue spectral region (400–500 nm) as the absorber layer thickness increases from 70 to 120 nm. This reduction is attributed to a loss mechanism where high-energy photons predominantly generate charge carrier pairs near the sun-facing side of the absorber, specifically close to the TiO_2_/Sb_2_S_3_ interface. Consequently, in thicker films, the collection of holes generated by these short-wavelength photons becomes limited by their diffusion length, preventing them from effectively reaching the hole transport layer (HTL). This phenomenon is consistent with a previous report.^45^ Conversely, lower-energy photons (wavelengths >550 nm) penetrate deeper into the absorber layer, generating charge carriers throughout the bulk. A distinct shoulder in the EQE spectrum around 650 nm suggests the presence of a beneficial optical spacer effect. This effect, commonly observed in thin-film solar cells, enhances the EQE at wavelengths above 650 nm, where the hole transport layer (HTL) P3HT typically does not absorb light.^28,46–48^ The magnitude of this gain is dependent on the thickness of both the absorber and the P3HT layers. The optical spacer effect significantly influences the EQE when the absorber thickness is around 100 nm or less; for thicker absorbers, most incident photons are absorbed before reaching the optical spacer layer, diminishing its impact. This interplay between absorber thickness and the optical spacer effect is clearly illustrated in the normalized EQE spectra (inset of Figure 3i). While light absorption across the 400–700 nm wavelength range generally increases with an increasing Sb_2_S_3_ absorber, absorption efficiency decreases otherwise. The photocurrent generation in Sb:S 1:6 devices is considerably lower compared to reference Sb:S 1:3 (90 nm) devices. This discrepancy may arise from increased deposition times associated with growing thicker films, which can lead to partial and “unwanted” crystallization (manifesting as dark brownish and dark green disc-like shapes in Figure 2d–h). Such uncontrolled crystallization does not follow proper growth procedures and can result in parasitic light absorption, thereby reducing the overall device performance. The optical band gap (*E*_g_), determined from (E*EQE)^2^ versus *hv* plots (Figure S3 in the Supporting Information), exhibits an inverse relationship with thinner Sb:S 1:6 films: the *E*_g_ of all samples was 1.72 eV except for Sb:S 1:6 (70 nm), where *E*_g_ was 1.74 eV.

These findings, improved FF ∼ 58% and *R*_Sh_ ∼ 1157 Ω cm^2^, suggest that solar cells fabricated from Sb:S 1:6 precursor molar ratio Sb_2_S_3_ films can potentially achieve comparable efficiencies to devices based on well-defined grain films, concomitantly indicating that grain size may not be the sole determinant of device performance. However, from the structural and morphological analysis, it is observed that the film quality of the Sb:S 1:6 needs optimization. The nonvisible grain boundaries and nondefined grains observed in Sb_2_S_3_ films especially in Sb:S 1:6 (40 cycles) 70 nm may be due to insufficient annealing time during the postdeposition treatment of the films or different deposition treatment, indicating it might be required to fully crystallize the films. Generally, inhomogeneous thickness, rough surface, incomplete coverage, or poor crystallinity can lead to increased surface recombination, light scattering losses, and hindered charge carrier transport and may ultimately result in lower device efficiency.^49,50^

In the development of ultrathin (<100 nm) film photoabsorbers, a fundamental trade-off exists between film thickness and device efficiency. As shown above, devices with both 70 and 120 nm absorbers demonstrated an average PCE of ∼4% with the maximum value of 4.5% achieved for 120 nm-based cells. However, the side effect of unwanted crystallization (see Supporting Information Figure S1a,b) in the 120 nm Sb_2_S_3_-based device implies challenges with reproducibility of high PCE. Considering these aspects, the device with 70 nm Sb_2_S_3_ is more prospective for further optimization, considering both reproducibility and its applicability niche (including semitransparency, tandem integration).^51,52^ For these combined reasons, the ultrathin Sb:S 1:6 (70 nm) film was chosen for further comprehensive optimization in the subsequent sections.

### Influence of Annealing Time and Sb:S Molar Ratio on USP-Deposited Sb_2_S_3_ Thin-Film Properties

To increase the film quality of the best-performing device of the 70 nm Sb:S 1:6 ultrathin film, postdeposition treatment annealing at 270 °C in N_2_ was varied ranging from 6 to 12 and 18 min. Figure 4a–d shows the surface view of the SEM images of the reference and the Sb:S with varied annealing time. Despite increasing the annealing time, well-defined grains were not observed in the Sb:S 1:6 films, as evidenced by surface SEM analysis (Figure 4b–d). From the XRD analysis, the crystalline quality and phases of the films are characterized. As shown in Figure 4e, the phase diffraction peaks of all the samples match well with the standard Sb_2_S_3_ pattern (ICSD 01-075-4012). The nondesired (*hk*0) planes, such as the (200) plane situated at 15.78°, which are observed in the reference Sb:S 1:3 (90 nm) and the Sb:S 1:6 (70 nm, 6 min) film, are absent in the Sb:S 1:6 (70 nm, 12 min) film but reemerge with increased intensity in the Sb:S 1:6 (70 nm, 18 min) film.

**Figure 4:**
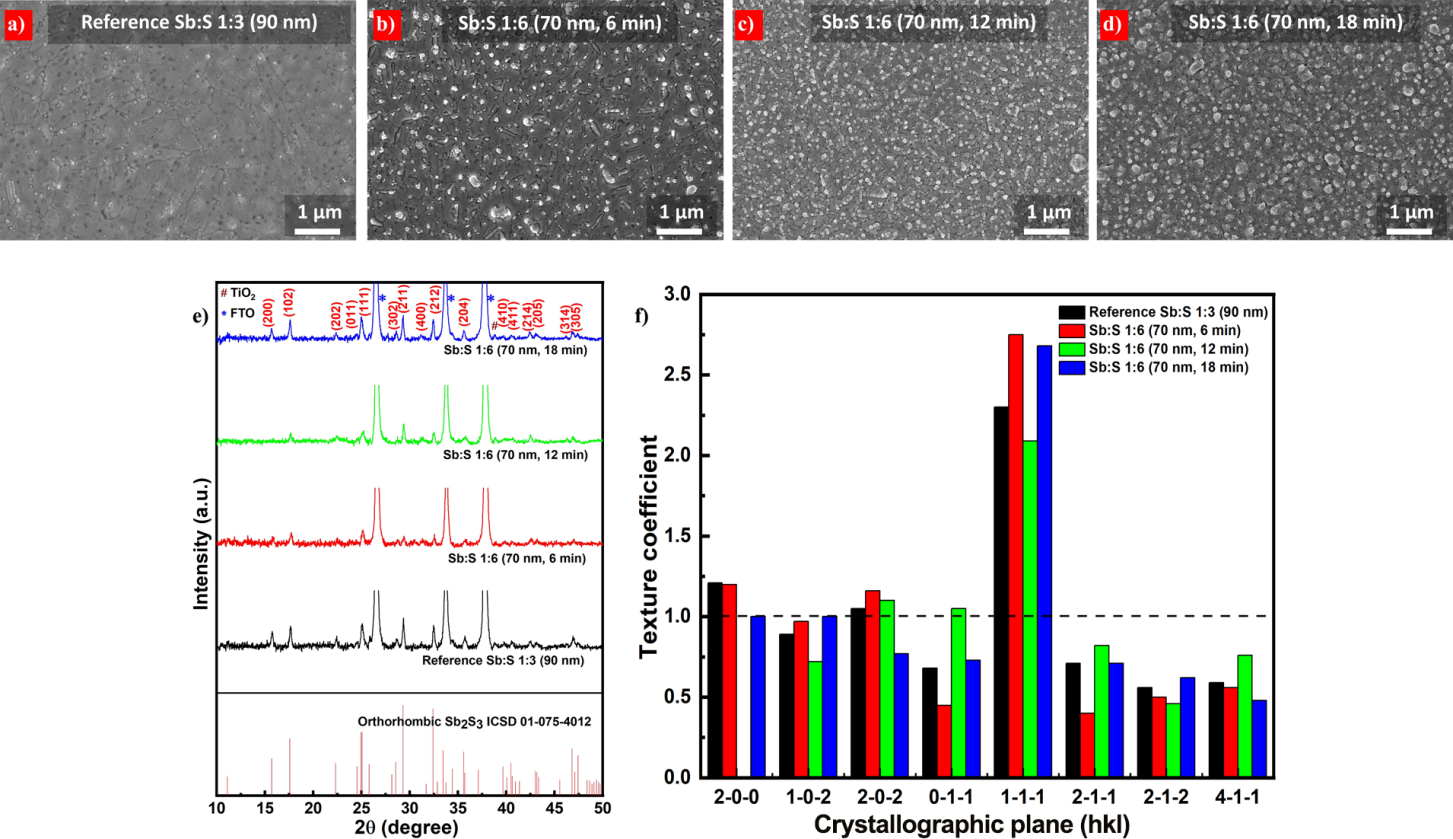
SEM images of (a) reference Sb:S 1:3 (90 nm), (b) Sb:S 1:6 (70 nm, 6 min), (c) Sb:S 1:6 (70 nm, 12 min), and (d) Sb:S 1:6 (70 nm, 18 min). (e) XRD patterns and (f) texture coefficients of the reference Sb:S 1:3 (90 nm) and Sb:S 1:6 (70 nm) thin films with varying annealing times.

XRD analysis revealed that increasing the annealing time of the Sb:S 1:6 (70 nm) sample from 6 to 18 min did not significantly alter the average Scherrer crystallite size of the dominant (*hk*1) peaks (011), (111), and (211) (situated at 24.64°, 25.08°, and 29.36°, respectively) as summarized in Table S4. From the TC analysis (Figure 4f), it can be observed that for the Sb:S 1:6 (70 nm, 12 min) sample, the texture coefficient corresponding to the (*hk*1) planes reaches or is close to unity, while the (200) plane disappears. This indicates that the 12 min of annealing in N_2_ seems to improve the crystallinity of the Sb:S 1:6 (70 nm) films. Moreover, from the optical properties (Figure S4), the absorption spectrum shown in Figure S4c of the Sb_2_S_3_ 1:6 (70 nm, 18 min) film shows weaker absorbance from 400 to 700 nm.

Considering that the Sb:S 1:6 (70 nm, 12 min) Sb_2_S_3_ ultrathin film shows the most promise from the point of view of structural properties, i.e., absence of the (200) plane and presence of dominant (*hk*1) planes, further analysis focused on this sample in comparison to the reference Sb:S 1:3 (90 nm) sample.

To deepen the phase content, crystalline quality, and potential defect formation, Raman spectra measured under 532 and 632.8 nm excitation wavelengths were analyzed. In both cases the spectra were measured in up to 16 points in each sample from the front (through the glass substrate) and from the back (at the thin-film surface), using a relatively high measurement spot (∼70 μm), allowing us to provide an average result in each measured spectrum. In the measured spectra of both the reference Sb:S 1:3 (90 nm) and Sb:S 1:6 (70 nm, 12 min) Sb_2_S_3_ thin films (Figure 5), only characteristic Raman peaks of the Sb_2_S_3_ phase were detected, showing the absence of a significant concentration of oxide phases and the absence of any concentration of pure Sb or S phases.

**Figure 5:**
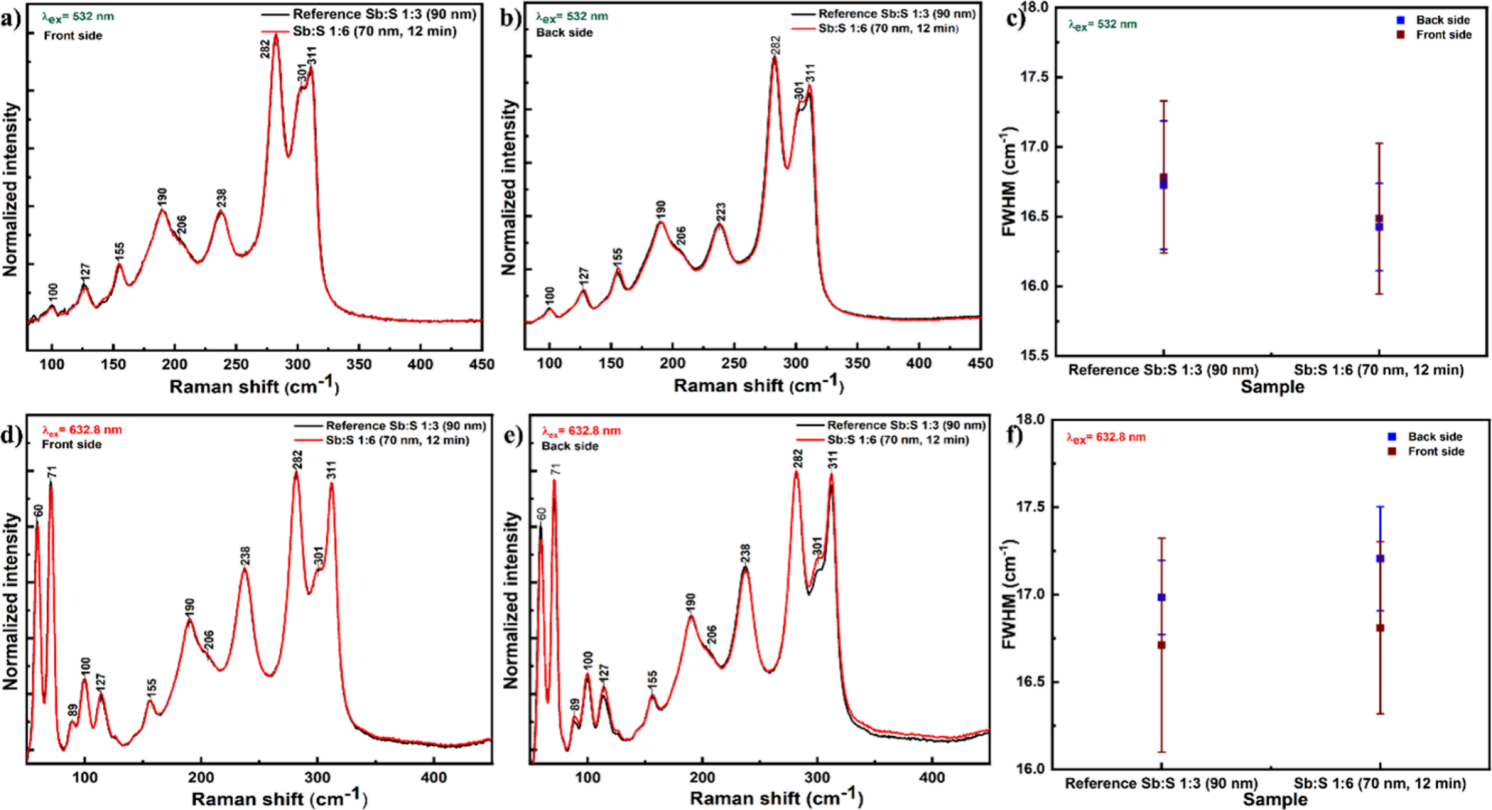
Raman spectra measured from the (a, d) front and (b, e) back side of the reference Sb:S 1:3 (90 nm) and Sb:S 1:6 (70 nm 12 min) samples under (a, b) 532 and (d, e) 632.8 nm excitation wavelength. (c, f) Full width at half-maximum (fwhm) of the main Raman peak at 282 cm^–1^ calculated for all measured spectra.

This was independent of the excitation wavelength and from the side of the samples from which the spectra were measured. The crystalline quality of the thin films was assessed by analysis of the full width at half-maximum (fwhm) of the Raman peaks. The calculated values of fwhm (Figure 5c,f) did not show a significant difference between two analyzed samples or when comparing results from the top or back of the samples. This confirms a comparable crystalline quality of the reference Sb:S 1:3 (90 nm) and Sb:S 1:6 (70 nm, 12 min) samples and similar crystalline quality through the thickness of the thin films. As discussed in a previous work on the vibrational properties of Sb_2_S_3_,^38^ the Raman peaks at lower frequencies (60–150 cm^–1^) can be assigned to the dominant Sb-related vibrations, while the peaks at higher frequencies (250–311 cm^–1^) can be assigned to the S-related vibrations. Changes in the relative intensity of one of these regions compared to the other indicate the domination of either Sb- or S-related vibrations and, thus, the increase of either Sb or S content in the measured samples. In the case of the reference and Sb:S 1:6 (70 nm, 12 min) thin films, the relative intensity of the peaks in the region of 60–130 cm^–1^ is increased compared to the stoichiometric monocrystalline samples used in ref 38, leading to the conclusion that all these samples are S-poor, and hence, the presence of corresponding point defects is possible.

To investigate the impact of precursor molar ratio on the elemental composition, X-ray photoelectron spectroscopy (XPS) and energy dispersive X-ray spectroscopy (EDS) analyses were performed on the reference Sb:S 1:3 (90 nm) and Sb:S 1:6 (70 nm, 12 min) samples. XPS analysis, utilizing the Sb 3d_3/2_ and S 2p core peaks (Figure S5a–d in the Supporting Information), revealed S/Sb ratios of 1.36 and 1.45 (Table 2) for the reference Sb:S 1:3 (90 nm) and Sb:S 1:6 (70 nm, 12 min) samples, respectively. Significantly, the Sb:S 1:6 (70 nm, 12 min) sample exhibited a S/Sb ratio closer to the stoichiometric composition compared to the reference Sb:S 1:3 (90 nm). These findings were corroborated by EDS analysis (Figures S7–S10 and Tables S5 and S6 in the Supporting Inormation), which yielded S/Sb ratios of 1.22 and 1.43 for the reference Sb:S 1:3 (90 nm) and Sb:S 1:6 (70 nm, 12 min) samples, respectively. These results demonstrate the successful tuning of film stoichiometry toward the desired 1.5 ratio by adjusting the precursor molar ratio.

**Table 2:** Elemental Composition of Reference Sb:S 1:3 (90 nm) and Sb:S 1:6 (70 nm, 12 min) Sb_2_S_3_ Films as Obtained from XPS (from Sb 3d_3/2_ and S 2p Core Levels) and EDS Studies

	XPS		EDS	
	Reference Sb:S 1:3 (90 nm)	Sb:S 1:6 (70 nm, 12 min)	Reference Sb:S 1:3 (90 nm)	Sb:S 1:6 (70 nm, 12 min)
Sb (at. %)	23.37	21.60	14.25	4.17
S (at. %)	31.54	31.42	17.44	5.95
S/Sb ratio	1.36	1.45	1.22	1.43

Figure 6a presents the XPS survey spectra, which reveal the presence of Sb, S, O, C, and N elements on the surfaces of both the reference Sb:S 1:3 (90 nm) and Sb:S 1:6 (70 nm, 12 min) samples. The presence of an O 2s core-level peak in the XPS spectrum of an Sb_2_S_3_ film indicates the presence of oxygen-related species, such as oxides (Sb_2_O_3_) or hydroxides.^53^ These oxygen-related species can adversely affect the film’s properties by forming oxide (Sb_2_O_3_) layers and modifying the electronic band structure.^54^ These defects can act as recombination centers and may reduce the carrier lifetime, ultimately degrading device performance. The high-resolution O 2s core-level spectra were fitted to determine the chemical state of oxygen at the surface (Figure 6b). Two peaks are identified at binding energies of ∼22.5 (mint blue) and 22.6 eV (green) in both samples. According to the reported literature, the binding energy peak at 22.6 eV corresponds to native oxygen, typical oxidation of a metal (Me–O), in this case Sb_2_O_3_, whereas the peak at 22.5 eV is non-native oxygen weakly adsorbed (adsb) on the surface.^55^ The presence of a C 1s peak in the XPS spectra indicates the adsorption of carbon on both surfaces, as shown in Figure S6a,b. The samples are then etched by Ar sputtering, and the carbon bonding ascribed to exposure to hydrocarbons and CO_2_ in ambient air is completely cleaned off, as shown in Figure S6c,d. After the etching, the adsb O 2s is also completely cleaned off in both samples, as shown in Figure 6c. In conclusion, the oxide content of the Sb:S 1:6 (70 nm, 12 min) sample can be easily etched as compared to the reference sample. As it has also been reported with the CBD deposition method, deep-level defects of Sb_2_S_3_ films can be effectively reduced by reducing the oxides introduced during the deposition process.^23,54,56^ Hence, the potential carrier recombination centers presented by the oxides are more reduced in the Sb:S 1:6 (70 nm, 12 min) films than in the reference Sb:S 1:3 (90 nm), having much influence on the solar cell performance of the Sb:S 1:6 (70 nm, 12 min) when postdeposition treatment is optimized. However, increasing the molar ratio of sulfur in solution to produce S-rich Sb_2_S_3_ samples may not be straightforward, or better Sb and S precursors are needed to fine-tune this approach.

**Figure 6:**
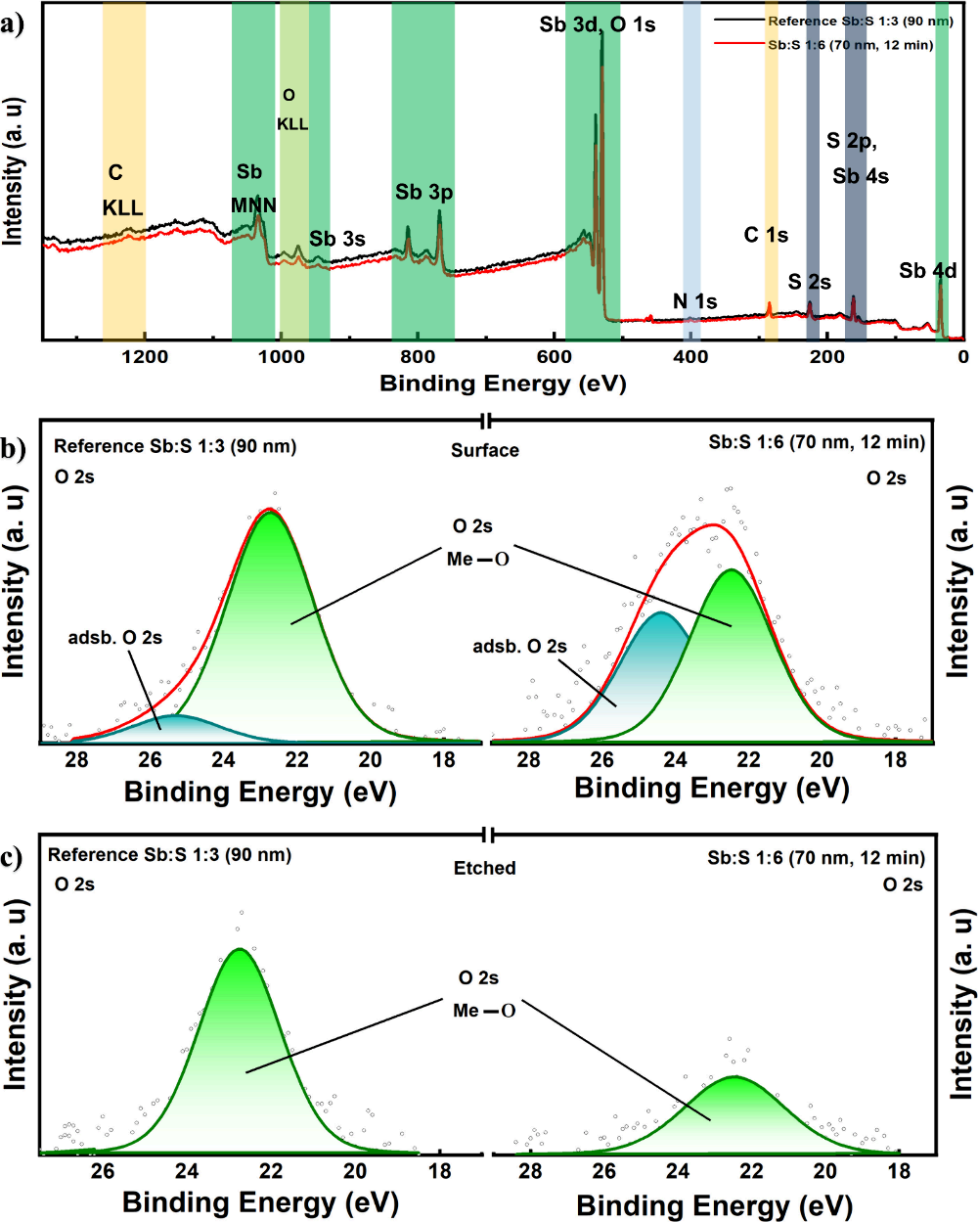
(a) XPS survey spectra of the reference Sb:S 1:3 (90 nm) and Sb:S 1:6 (70 nm, 12 min) samples. (b, c) High-resolution O 2s core-level XPS spectra of the surface and etched regions of the reference Sb:S 1:3 (90 nm) and Sb:S 1:6 (70 nm, 12 min) samples.

### Influence of Annealing Time and Sb:S Ratio on the Electrical Properties of USP-Deposited Sb_2_S_3_ Solar Cells

To investigate the impact of postdeposition annealing time on device performance, the Sb:S 1:6 (70 nm) ultrathin film was annealed for 6, 12, and 18 min to optimize the postdeposition treatment. These samples were then integrated into glass/FTO/Sb_2_S_3_/P3HT/Au solar cell structures. Figure 7a,b shows the light and dark *J*–*V* curves of the best-performing devices from each group and the reference Sb:S 1:3 (90 nm), while Figure 7c–h displays the corresponding statistical plots. Table 3 summarizes the photoelectric parameters extracted from six devices for each sample. The Sb:S 1:6 (70 nm, 12 min) device exhibited the highest PCE of 5.3%, with a *V*_OC_ of 661 mV, a *J*_SC_ of 13 mA cm^–2^, and an FF of 62%. Compared to the PCE of the reference Sb:S 1:3 (90 nm) device (4.7%), the Sb:S 1:6 (70 nm, 6 min) device (4.3%), and the Sb:S 1:6 (70 nm, 18 min) device (4.0%), the PCE of the Sb:S 1:6 (70 nm, 12 min) device increased by 13%, 23%, and 32%, respectively. The efficiencies extracted from the Sb:S 1:6 (70 nm, 12 min) devices are within a narrow distribution range of 4.8–5.3%, shown in Figure 7f, indicating high homogeneity of the process.

**Figure 7:**
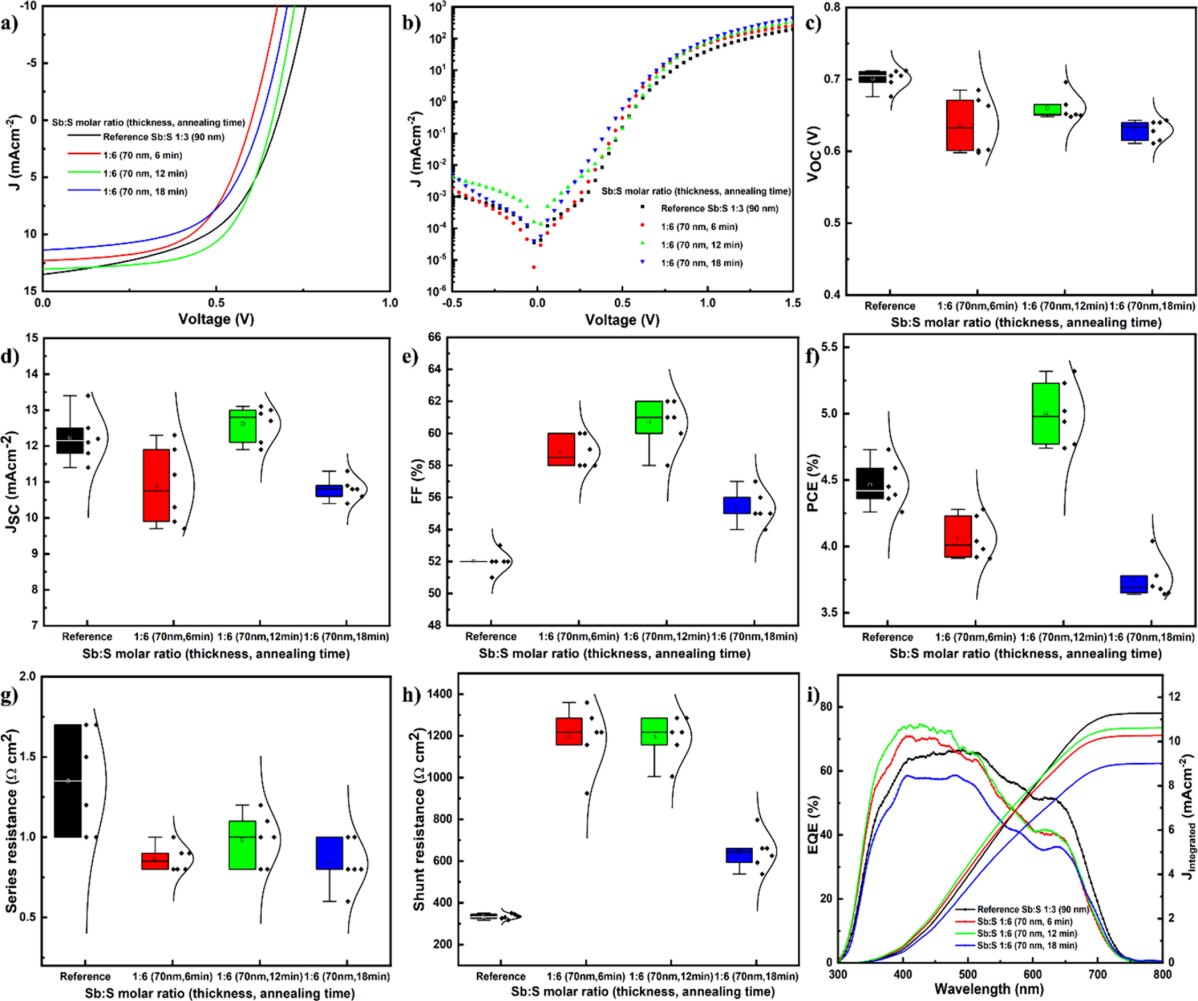
(a) Illuminated and (b) dark *J*–*V* curves of highest-performing devices and statistical analysis of (c) *V*_OC_, (d) *J*_SC_, (e) FF, (f) PCE, (g) series resistance, and (h) shunt resistance for solar cells measured under AM 1.5G illumination as a function of Sb:S precursor molar ratio and varying annealing times. (i) External quantum efficiency (EQE) and integrated *J*_SC_ curves of the best-performing cells based on Sb:S precursor molar ratio and varying annealing times. The reference sample is Sb:S 1:3 (90 nm, 6 min).

**Table 3:** Photovoltaic Performance Parameters of the Reference Sb:S 1:3 and Sb:S 1:6 (70 nm) Molar Ratio Samples Annealed for 6 and 12 min, Respectively, Measured under AM 1.5G Illumination Conditions

		*J*_SC_ (mA cm^–2^)						
Sb:S (annealing time)	*V*_OC_ (mV)	*I*–*V*	EQE	FF (%)	PCE (%)	*J*_0_ (mA cm^–2^)	*R*_S_ (Ω cm^2^)	*R*_Sh_ (Ω cm^2^)
Reference 1:3 (70 nm)	676	13.4	11.3	52.0	4.7	3.59 × 10^–5^	1.5	345
	700 ± 13^a^	12.2 ± 0.7		52 ± 1.0	4.5 ± 0.2		1.4 ± 0.3	334 ± 12
1:6 (70 nm, 6 min)	602	12.3	10.3	58.0	4.3	5.68 × 10^–6^	1	1157
	637 ± 40	11.0 ± 1.1		58.8 ± 1.0	4.1 ± 0.1		0.9 ± 0.1	1193 ± 148
1:6 (70 nm, 12 min)	661	13.0	10.6	62.0	5.3	8.75 × 10^–5^	0.8	1217
	660 ± 18	12.6 ± 0.5		60.1 ± 1.5	5 ± 0.2		1 ± 0.2	1194 ± 104
1:6 (70 nm, 18 min)	628	11.3	9.0	57.0	4.0	5.67 × 10^–5^	0.8	797
	629 ± 14	10.8 ± 0.3		55.3 ± 1.0	3.7 ± 0.2		0.8 ± 0.2	645 ± 87

a Average and standard deviation.

The 12 min annealing time postdeposition treatment may be beneficial for improving the film quality of the devices, yet there are no visible grain boundaries or any defined grains in the film (Figure 4c). The Sb_2_S_3_ 1:6 (70 nm, 6 min) and Sb_2_S_3_ 1:6 (70 nm, 18 min) films show poor performances. This is most likely because the 6 min annealing time is too short for complete crystallization of the amorphous phase. On the other hand, the 18 min treatment is too long, which may start to evaporate the Sb_2_S_3_ film (reducing its thickness), decreasing its absorption magnitude in the visible region as shown in Figure S4c in the Supporting Information, and may also create microcracks in the Sb_2_S_3_ film, thus creating current leakage pathways and decreasing *R*_Sh_ from 1.2 to 0.7 kΩ cm^2^. Absorption efficiency is generally reduced for the ultrathin Sb:S 1:6 (70 nm) films as compared to the reference Sb:S 1:3 (90 nm); this is worsened in the 18 min annealed Sb:S 1:6 (70 nm, 18 min) film as we start to lose the Sb_2_S_3_ material because of longer annealing time. According to the statistical plots in Figure 7c–h, the improvement of the Sb:S 1:6 (70 nm, 12 min) device is mainly due to the FF (62%). According to the literature, the FF of best-performing Sb_2_S_3_ solar cells by the spray pyrolysis deposition method mostly ranges from 50 to 55%;^28,31,57^ the record cell which is by CBD has an FF of 60%.^15^ The low *R*_S_ (0.8 Ω cm^2^) and high *R*_Sh_ (1.2 kΩ cm^2^) are the main reasons of the improved FF (62%), which mainly depend on bulk defects and p–n junction or interface contact quality.^58^

The overall EQE response (Figure 7i) generally aligns with previous observations. However, a distinct behavior is noted for high-energy photons (400–500 nm): the EQE response in this region is enhanced for Sb:S 1:6 (70 nm, 12 min) devices annealed for 12 min but significantly degrades when the annealing duration is extended to 18 min. This improvement at 12 min of annealing suggests an optimization of the Sb:S 1:6 (70 nm) film quality, likely facilitating more efficient photogenerated charge collection at the TiO_2_/Sb_2_S_3_ interface. Consistent with established principles, the prominent EQE shoulder observed at around 650 nm indicates the presence of an optical spacer effect, whose magnitude is critically dependent on the absorber layer thickness. Conversely, the observed reduction in charge extraction efficiency for the Sb:S 1:6 (70 nm, 18 min) device subjected to 18 min of annealing can be attributed to several factors induced by prolonged thermal treatment. Extended annealing may lead to decreased film thickness (as shown in the transmittance and absorption spectra in Figure S4a–c). Furthermore, it is plausible that sublimation of Sb_2_S_3_ begins to occur at this extended annealing time, resulting in a reduction of the active film thickness and, consequently, diminished charge extraction, particularly impacting the EQE response around 650 nm. Concurrently, the band gap values, determined from the EQE spectra, exhibited a slight decrease from 1.75 to 1.73 eV with increasing annealing time (Figure S11).

Current–voltage temperature (*I*–*V*–*T*)-dependent measurements are further carried out to investigate the recombination behavior of the devices both in the dark and under illuminated conditions, as shown Figure 8a,b. The *V*_OC_–*T* dependence for the reference and the best-performing Sb:S 1:6 (70 nm, 12 min) with optimized annealing time is first analyzed based on eq 2:^41^

2 $$V_{\mathrm{OC}}=\frac{E_{A}}{q}-\frac{{Ak}_{B}T}{q}\;\ln\left(\frac{J_{0}}{J_{\mathrm{ph}}}\right)$$

where *T* is temperature, *k*_B_ is the Boltzmann constant, *A* is the ideality factor, *J*_0_ is the reverse saturation diode current prefactor, *J*_ph_ is the photocurrent density, and *E*_A_ is the activation energy.

**Figure 8:**
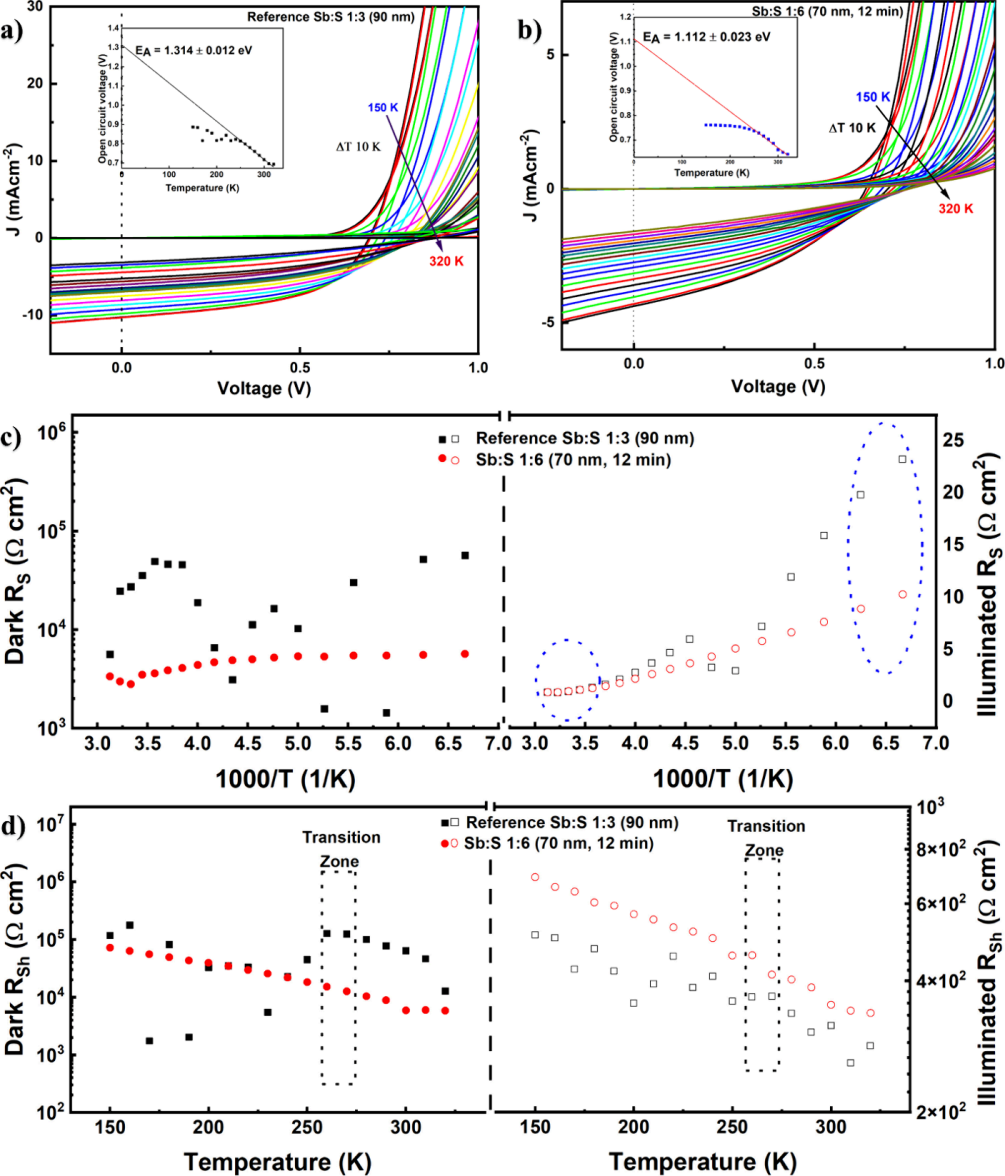
Temperature-dependent current–voltage (*J*–*V*) characteristics of (a) reference Sb:S 1:3 (90 nm) and (b) Sb:S 1:6 (70 nm, 12 min) solar cells. Insets: temperature dependence of *V*_OC_ with extrapolated activation energy (*E*_A_) at 0 K. (c) Temperature-dependent series resistance (*R*_S_) and (d) shunt resistance (*R*_Sh_) extracted from dark and light *J*–*V* measurements.

The temperature-dependent *V*_OC_ exhibits an initial increase at low temperatures for both samples, which may be attributed to the suppression of the recombination processes. Below 220 K, the Sb:S 1:6 (70 nm, 12 min) sample reaches a plateau in *V*_OC_, while the reference Sb:S 1:3 (90 nm) sample shows slight variations. Extrapolation of the linearly increasing portion of the *V*_OC_–temperature curves to 0 K yields activation energies of 1.31 and 1.11 eV for the reference Sb:S 1:3 (90 nm) and Sb:S 1:6 (70 nm, 12 min) samples, respectively. These values are lower than the band gap (*E*_g_ ∼ 1.72 eV) of the Sb_2_S_3_ absorber layer shown in Figure S11, indicating that interface recombination is the dominant mechanism limiting *V*_OC_ in both devices. The case is slightly worse in the Sb:S 1:6 (70 nm, 12 min) device, as its activation energy is 18% lower than that of the reference Sb:S 1:3 (90 nm), hence further indicating that increasing the molar ratio may not suppress interface recombination significantly. However, both devices show reduced surface recombination and have comparable values with the literature.^59^ For both devices, the dominant contributor to the temperature-dependent efficiency behavior is *J*_SC_ and FF behavior with temperature. These parameters decrease with decreasing temperature (Figure S12a,b). Typically, these device parameters exhibit a minimal response to temperature in an ideal solar cell. Cooling from 300 to 150 K resulted in a substantial decrease in device performance, with *J*_SC_, FF, and PCE decreasing by 76%, 87%, and 80%, respectively, relative to their room temperature values. Notably, the Sb:S 1:6 (70 nm, 12 min) devices demonstrated a significantly improved temperature stability, with *J*_SC_, FF, and PCE decreasing by only 27%, 31%, and 17%, respectively, relative to their room temperature values. The temperature-dependent *R*_S_ analyzed in Figure 8c for both devices in the dark and under illumination shows a linear increase with decreasing temperature especially below 220 K. The Sb:S 1:6 (70 nm, 12 min) sample exhibited significantly lower dark *R*_S_ (by 1 order of magnitude) compared to the reference Sb:S 1:3 (90 nm) sample, especially at temperatures below 220 K. The transition of the Sb:S 1:6 (70 nm, 12 min) device to a resistive state at temperatures below 220 K indicates a sudden decrease in capacitance; this may be due to device degradation caused by the carrier freeze-out effect, which is typical in materials with deep defect levels or incomplete lattice matching at interfaces.^37,60,61^ Additionally, undesirable phase transitions within one or more device layers may contribute to this behavior. As the temperature decreases from 320 to 260 K, the dark *R*_Sh_ (Figure 8d) increases linearly in both devices, though the dark *R*_Sh_ of Sb:S 1:6 (70 nm, 12 min) is 1 order of magnitude lower than that of the reference in this region. There is a sharp decrease in dark *R*_Sh_ at temperatures below 260 K for the reference Sb:S 1:3 (90 nm) sample, and the same values begin to be obtained with the Sb:S 1:6 (70 nm, 12 min) device. Between 270 and 260 K, a thermally activated phase transition process occurs, and its *R*_Sh_ jumps to a transition zone. Under illumination conditions, the *R*_Sh_ of the Sb:S 1:6 (70 nm, 12 min) increased by a few margins higher than that of the reference from 320 to 150 K. The reference sample shows even higher fluctuations of *R*_Sh_ values after the transition zone, but the *R*_Sh_ of the Sb:S 1:6 (70 nm, 12 min) is unaffected and increases linearly. The abrupt change observed in the reference Sb:S 1:3 (90 nm) sample at the transition zone is likely attributed to the known phase transition of P3HT at 263 K.^27^ Additionally, the nonlinear temperature dependence of the Sb_2_S_3_ dielectric permittivity near 270 K may contribute to this anomalous behavior.^27^ However, the Sb_2_S_3_ 1:6 (70 nm, 12 min) sample exhibits more stable behavior, suggesting a more rigid structure. As previously reported,^27^ the overall behavior of the shunt resistance is influenced by the synthesis history of the absorber layer.

To determine the depletion width (*W*_0_), charge carrier concentration (*N*_D_), and built-in potential (*V*_bi_) of the devices, capacitance–voltage measurements were performed. A parallel plate capacitor model was employed to describe the abrupt p–n heterojunction capacitance, and the impedance behavior (Figure 9a) was recorded at a frequency of 11.8 kHz. The *W*_0_, *N*_D_, and *V*_bi_ were estimated according to eqs 3 and 4:^62^

3 $$W_{0}=\varepsilon \varepsilon_{0}\frac{A}{C}$$

4 $$N_{D}=-\frac{C^{2}}{q\varepsilon \varepsilon_{0}A^{2}}\left(V_{\mathrm{bi}}-V\right)$$

where *C* is the capacitance, *W*_0_ is the depletion width, *A* is the area of the device, ε is the relative permittivity of Sb_2_S_3_, ε_0_ is the vacuum permittivity, *V*_bi_ is the built-in potential, *V* is the bias voltage, and *q* is the elementary charge. Interestingly, the Sb:S 1:6 (70 nm, 12 min) device, despite having a thinner (70 nm) Sb_2_S_3_ photoabsorber layer, exhibits a wider *W*_0_ (48.4 nm) compared to the 90 nm thick reference Sb:S 1:3 device (90 nm). While a wider *W*_0_ can contribute to increased *R*_S_, the Sb:S 1:6 (70 nm, 12 min) device maintains a low *R*_S_ of 0.8 Ω cm^2^. This trade-off between *W*_0_ and *R*_S_ suggests that the device offers enhanced light absorption and a stronger electric field, which may facilitate efficient charge separation and reduced recombination losses. However, the Sb:S 1:6 (70 nm, 12 min) device produces a slightly lower *V*_OC_ (661 mV) compared to the reference Sb:S 1:3 (90 nm) device (676 mV), which has a higher *R*_S_ (1.5 Ω cm^2^) and a narrower *W*_0_ (39 nm). This discrepancy may be attributed to the complex interplay among several factors, including interface quality, defect density, and carrier transport properties.

**Figure 9:**
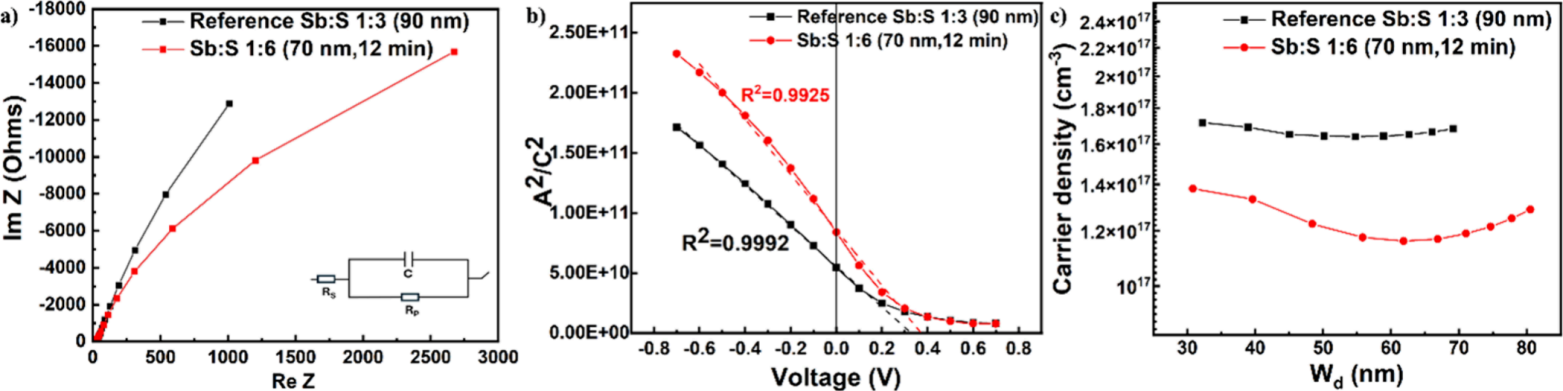
Electrical characterization. (a) Impedance spectroscopy (Nyquist plots), (b) capacitance–voltage (Mott–Schottky) measurements, and (c) charge carrier density (*N*_D_) profiles through the depletion region (*W*_0_) of reference Sb:S 1:3 (6 min) and Sb:S 1:6 (12 min) solar cells.

The *V*_bi_ is a critical parameter that acts as an energy barrier, preventing spontaneous recombination of charge carriers and enabling efficient charge separation and collection at the respective electrodes. The Sb:S 1:6 (70 nm,12 min) device displayed a *V*_bi_ of 0.368 V (Figure 9b), slightly higher than the 0.328 V of the reference device as presented in Table 4. This value is comparable to those reported in the literature.^59^ As one should expect, a higher *V*_bi_ should complement the *V*_OC_, but this effect is different for the case of Sb:S 1:6 (70 nm, 12 min). As seen from the *N*_D_ obtained using eq 4, which gives much better precision at short-circuit conditions (*V* = 0), the Sb:S 1:6 (70 nm, 12 min) produced an *N*_D_ of 1.23 × 10^17^ cm^–3^, on the same order of magnitude but a few margins lower than that of reference Sb;S 1:3 (90 nm), 1.69 × 10^17^ cm^–3^, as presented in Table 4. As the reverse bias polarization increases (*V* < 0), the *W*_0_ expands, enabling the determination of the *N*_D_ deeper into the layer by using eq 4. Figure 9c illustrates the behavior of *N*_D_ as a function of increasing *W*_0_ for both devices. While the *N*_D_ values for both devices are on the same order of magnitude at greater depths, the Sb:S 1:6 (70 nm, 12 min) device consistently exhibits slightly lower values. For the Sb:S 1:6 (70 nm, 12 min) device, *N*_D_ initially peaks at lower depletion widths and then decreases sharply until reaching 62 nm, after which it increases linearly.

**Table 4:** Optoelectronic Properties (Built-In Potential *V*_bi_, Depletion Width *W*_0_, and Carrier Density *N*_D_) of Reference Sb:S 1:3 (90 nm) and Sb:S 1:6 (70 nm, 12 min)

Sample	*V*_bi_ (V)	*W*_0_ (nm)	*N*_D_ (cm^–3^)
Reference Sb:S 1:3 (90 nm)	0.328	39.0	1.69 × 10^17^
Sb:S 1:6 (70 nm, 12 min)	0.368	48.4	1.23 × 10^17^

In contrast, the reference device shows a more gradual decrease in *N*_D_ until 55 nm, followed by a slight rebound. However, since the carrier density is on the same order of magnitude in both devices, it is unlikely to have a significant impact on device performance. The observed disparities in cell performance can be attributed to the combination of higher *W*_0_ and *V*_bi_ values in the Sb:S 1:6 (70 nm, 12 min) device. Counterintuitively, increasing the sulfur molar ratio in the precursor solution may be expected to suppress defect formation, thereby increasing the carrier concentration. However, this approach is not straightforward. Zhu’s group^56^ demonstrated this by coevaporating sulfur to produce 100 nm S-rich Sb_2_S_3_ films. Despite the S-rich environment, their resulting films exhibited a lower carrier concentration (1.16 × 10^15^ cm^–3^) and a lower PCE (5.8%) compared to expectations. They attributed the lower carrier concentration to the formation of interstitial S defects, which can act as recombination centers, similar to S vacancies and Sb_S_ antisites.^56^ In this work, the comparable carrier concentration of the Sb:S 1:6 (70 nm, 12 min) and the reference Sb:S 1:3 (90 nm) suggests that increasing the sulfur precursor ratio may have unintended consequences on the material’s electronic properties. To gain a deeper understanding of this phenomenon and potentially improve device performance, a comprehensive density functional theory (DFT) study of Sb_2_S_3_ or the exploration of alternative sulfur precursors may be beneficial. However, the ultrathin film Sb:S 1:6 (70 nm, 12 min) shows a much improved depletion width with a better trade-off *R*_S_, which contributed to its high performance.

## Conclusions

In this study, we present a novel industrially scalable fabrication protocol for high-quality ultrathin Sb_2_S_3_ films with enhanced solar cell performance. The core innovation lies in manipulating the precursor molar ratio of antimony trichloride:thiourea (SbCl_3_:TU) by incorporating excess TU, targeting a unique Sb:S 1:6 ratio during deposition. Comprehensive analysis reveals the impact of this ratio on film properties and device performance. X-ray photoelectron spectroscopy (XPS) and energy dispersive X-ray spectroscopy (EDS) confirm a S/Sb atomic percentage ratio of 1.45 and 1.43, respectively, for the Sb:S 1:6 ratio, demonstrating improved stoichiometry compared to the established Sb:S 1:3 ratio (1.36 and 1.22, respectively). Despite this improvement, multiwavelength Raman spectroscopy indicates persistent sulfur deficiency in both ratios. X-ray fluorescence (XRF) confirms uniform film growth and a homogeneous thickness for both ratios. While the precursor ratio manipulation does not significantly affect the band gap, crystallinity, or carrier concentration, it substantially alters the film growth rate, morphology, and charge transport properties. Notably, the ultrathin Sb:S 1:6 (70 nm, 12 min) leads to a wider *W*_0_ ∼ 48.4 nm and higher *V*_bi_ ∼ 0.368 V, resulting in enhanced device performance. Ultrathin Sb_2_S_3_ (70 nm, 12 min) films fabricated with the 1:6 ratio achieve a high fill factor of 62%, comparable to reported record cells with significantly thicker films, and a PCE of 5.3%. This performance highlights the potential of this technique for realizing high-performance Sb_2_S_3_ solar cells. These findings demonstrate the effectiveness of precursor molar ratio engineering for optimizing Sb_2_S_3_ solar cell performance, paving the way for scalable, cost-effective, and high-performance devices and contributing significantly to the advancement of next-generation solution-processed photovoltaics.

## Experimental Section

### Materials

Thiourea (CH_4_N_2_S, 99.0%, Sigma-Aldrich), antimony chloride (SbCl_3_, 99.0 wt %, Sigma-Aldrich), methanol (99.9 vol %, Honeywell), ethanol (96.6 vol %, Estonian Spirit Ltd.), acetylacetone (99 vol %, Thermo Scientific), titanium tetraisopropoxide (98 vol %, Thermo Scientific), isopropanol (99.8 vol %, Honeywell), poly­(3-hexyl-2-5-diylthiophene) (50–100 kDa, >90% regioregular, Sigma-Aldrich), chlorobenzene (99.9 wt %, Acros), and acetone (99.9 vol %, Honeywell) were used, and all of the chemicals were used as received.

### Device Fabrication

Soda-lime glass substrates coated with a 200 nm fluorine-doped tin oxide (FTO) layer were cleaned sequentially with soap, deionized water, acetone, and isopropyl alcohol in an ultrasonic bath for 10 min each, followed by drying in air at 105 °C.^31^ A dense, compact TiO_2_ anatase layer (30 nm) was deposited onto the FTO-coated glass substrates using ultrasonic spray pyrolysis (USP) as the electron transport layer (ETL).^31,63,64^ A 0.2 M solution of titanium­(IV) isopropoxide (TTIP) and acetylacetone in ethanol was used as the precursor solution. The deposition was carried out at a substrate temperature of 340 °C and a spray rate of 2.5 mL/min, followed by annealing at 450 °C for 30 min in air. Amorphous Sb_2_S_3_ thin films were deposited onto the TiO_2_-coated substrates using USP. The precursor solutions were prepared by dissolving antimony­(III) chloride (SbCl_3_) and thiourea (SC­(NH_2_)_2_) in methanol at a 1:3 molar ratio for the reference cell and a 1:6 molar ratio for the experimental cells. The reference cell was deposited for 40 cycles under the same conditions as those used for our record cell.^28^ The experimental cells were deposited at varying spray cycles from 40 to 70 cycles at 185 °C and subsequently crystallized by thermal annealing at 270 °C for 6 min in a nitrogen atmosphere. Regioregular poly­(3-hexylthiophene) (P3HT) was used as the hole transport layer (HTL). A 2% w/w solution of P3HT in chlorobenzene was spin-coated onto the Sb_2_S_3_ layer and annealed at 150 °C for 5 min under nitrogen.^65^ Finally, a gold counter electrode was thermally evaporated onto the P3HT layer to complete the device fabrication.

### Measurements and Characterization

The as-deposited and annealed Sb_2_S_3_ thin films of the glass/FTO/TiO_2_/Sb_2_S_3_ stack underwent rigorous structural and phase analysis. As described previously, Cu Kα (λ = 1.54 Å, 40 kV, 40 mA) X-ray diffraction (Rigaku Ultima IV) unveiled the crystal structure and phase composition, while micro-Raman spectroscopy (Horiba Labram HR 800, 532 nm He:Ne Laser, backscattering) provided further insights into crystalline quality and potential traces of different secondary phases at room temperature.^37^ Additional Raman spectra were measured at 532 and 632.8 nm excitation wavelengths. The spectra were collected by using a Horiba FHR 640 monochromator coupled with a CCD detector. The measurements were performed in the backscattering configuration through the special probe-head designed in IREC. Nd:YAG solid state (532 nm) and He:Ne gas (632.8 nm) lasers were used as excitation sources. Surface analysis of the thin-film stack utilized high-resolution SEM (Helios NanoLab 600, FEI Company) and an energy dispersive X-ray spectrometer (Bruker ESPRIT 1.8, 7 kV) system to examine the surface morphology and elemental composition as per previously detailed experiments.^27,57^ The atomic concentrations as well as the thickness of the different layers were acquired by X-ray fluorescence at 50 kV with a Ni10 filter (Fischerscope X-ray XDAL 237 SSD). Additionally, optical characterization was performed using a UV–vis–NIR spectrophotometer (Jasco V-670) equipped with an integrating sphere and an air reference, providing insight into the light absorption and reflection properties of the sample.

X-ray photoelectron spectroscopy (XPS) was employed to analyze the surface composition and chemical state of the heat-treated Sb_2_S_3_ films. A PSP Vacuum Technology hemispherical electron-energy analyzer equipped with monochromated Al Kα radiation (*hν* = 1486.6 eV) was used to characterize the reference and optimal experimental films. The spectrometer was calibrated using a clean polycrystalline silver foil Ar^+^ sputtered under vacuum operating with an overall resolution of ±0.1 eV.^66^ High-resolution XPS spectra were fitted with Voigt functions to accurately determine the peak shapes and positions. For a more comprehensive analysis, additional XPS measurements were performed using a SPECS PHOIBOS 150 2D-DLD hemispherical electron-energy analyzer equipped with a Mg Kα (*hν* = 1253.6 eV) source.^67^ High-resolution XPS spectra were collected with a pass energy of 50 eV and a step size of 0.1 eV. The spectra were analyzed using CasaXPS software with Shirley background subtraction and Voigt-like symmetric Lorentzian peak fitting. The binding energies of the Sb 3d, Sb 4d, S 2p, and O 2s core levels were calibrated using the adventitious carbon C 1s peak at 284.5 eV.^68^

Current–voltage (*I*–*V*) measurements were performed using a factory-calibrated solar simulator (Wavelabs LS2) under AM 1.5G, 100 mW/cm^2^ conditions at room temperature. Temperature-dependent *I*–*V* curves were obtained by using a closed-cycle helium cryostat (Janis CCS-150) and a calibrated halogen lamp. External quantum efficiency (EQE) spectra were measured by using a monochromated light source (Newport 300 W xenon lamp with a Cornerstone 260 monochromator) at zero bias voltage. A digital lock-in detector (Merlin) and a calibrated silicon reference detector were employed for accurate measurements. The short-circuit current density (*J*_SC_) was determined by integrating the EQE spectra under AM 1.5G conditions.

Impedance measurements were performed by using a Biologic impedance analyzer and EC-LAB software. A constant bias signal with a 20 mV perturbation was applied over a frequency range of 5 GHz to 300 Hz. To minimize signal interference, the measurements were conducted within a Faraday chamber. The experimental data were fitted to an equivalent circuit model to extract relevant parameters.

## Supplementary Material



## Notes

Summary of deposition cycles and corresponding film thicknesses; XRD patterns and Raman spectra of as-deposited samples with varying thicknesses; Raman spectra measured under different excitation conditions and fwhm of main Raman peak at 282 cm^–1^ of the annealed films with varying thicknesses; summary of crystallite size based on (200), (011), and (211) planes of the samples with varying thicknesses; UV–vis spectra; band gaps obtained from (*E**EQE)^2^ versus *hv* plots; 2D XPS data; SEM images; tabulated atomic concentrations from EDS; and *I*–*V*–*T* data of solar cells (PDF)
