# Supplementary material for: Influence of Sulfur Source on Growth of In-Air Sprayed Ultrathin Film Sb2S3 for Enhanced Solar Cell Performance
Source: ACS Appl Mater Interfaces. 2025 Nov 11;17(47):64753–70. doi: 10.1021/acsami.5c17869 (PMC12673516; doi:10.1021/acsami.5c17869)
Supplement: Supplementary file 1 [file am5c17869_si_001.pdf]

Supporting Information

Influence of Sulfur Source on Growth of in-Air Sprayed Ultrathin Film Sb_2S_3 for Enhanced Solar Cell Performance

Ernest A. Asare[a], Atanas Katerski[a], Merike Kriisa[a], Raavo Josepson[b], Victoria Rotaru[c,d], Maxim Guc[c], David Payno[c], Alejandro Navarro-Güell[g,h], Raitis Grzibovskis[f], Aivars Vembris[f], Alejandro Pérez-Rodríguez[c,e], Edgardo Saucedo[g,h], Nicolae Spalatu[a]*, Malle Krunks[a]*, Ilona Oja Acik[a]**

[a] Department of Materials and Environmental Technology, Tallinn University of Technology, Ehitajate tee 5 Tallinn, Estonia

[b] Division of Physics, Tallinn University of Technology, Ehitajate tee 5 Tallinn, Estonia

[c] Catalonia Institute for Energy Research (IREC), Jardins de les Dones de Negre 1, Sant Adrià de Besòs, 08930 Barcelona, Spain

[d] Facultat de Fisica, Universitat de Barcelona (UB), C. Marti i Franques 1-11, 08028 Barcelona, Spain

[e] Departament d'Enginyeria Electronica i Biomedica, IN2UB, Universitat de Barcelona, C. Marti i Franques 1-11, 08028 Barcelona, Spain

[f] Institute of Solid State Physics, University of Latvia, Kengaraga Str. 8, Riga, LV-1063, Latvia

[g] Electronic Engineering Department, Universitat Politècnica de Catalunya (UPC), Av. d'Eduard Maristany 16, 08019 Barcelona, Spain

[h] Barcelona Center for Multiscale Science and Engineering, Universitat Politècnica de Catalunya (UPC), Av. d'Eduard Maristany 16, 08019 Barcelona, Spain

*Corresponding author.

E-mail address: Email: nicolae.spalatu@taltech.ee (N. Spalatu), ilona.oja@taltech.ee (I. Oja Acik), malle.krunks@taltech.ee (M. Krunks), ernest.asare@taltech.ee (E. A. Asare)

Table S1. Summary of USP deposition cycles and corresponding film thicknesses.

**Deposition cycles**	**Thickness (nm)**

Reference Sb:S 1:3 (40 cycles)	90
Sb:S 1:6 (40 cycles)	70
Sb:S 1:6 (50 cycles)	90
Sb:S 1:6 (60 cycles)	100
Sb:S 1:6 (70 cycles)	120

Table S2. Summary of Grain sizes by Feret diameter method for the USP deposited cycles and corresponding film thicknesses.

Deposition cycles	Grain Size (μm)
Reference Sb:S 1:3 (40 cycles)	3.88 ± 0.72
Sb:S 1:6 (40 cycles)	--
Sb:S 1:6 (50 cycles)	6.70 ± 1.11
Sb:S 1:6 (60 cycles)	4.04 ± 1.20
Sb:S 1:6 (70 cycles)	7.27 ± 0.15 / 3.27 ± 0.90

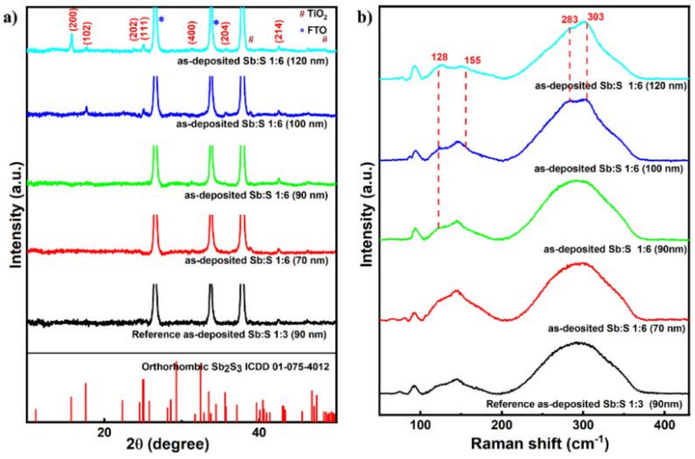

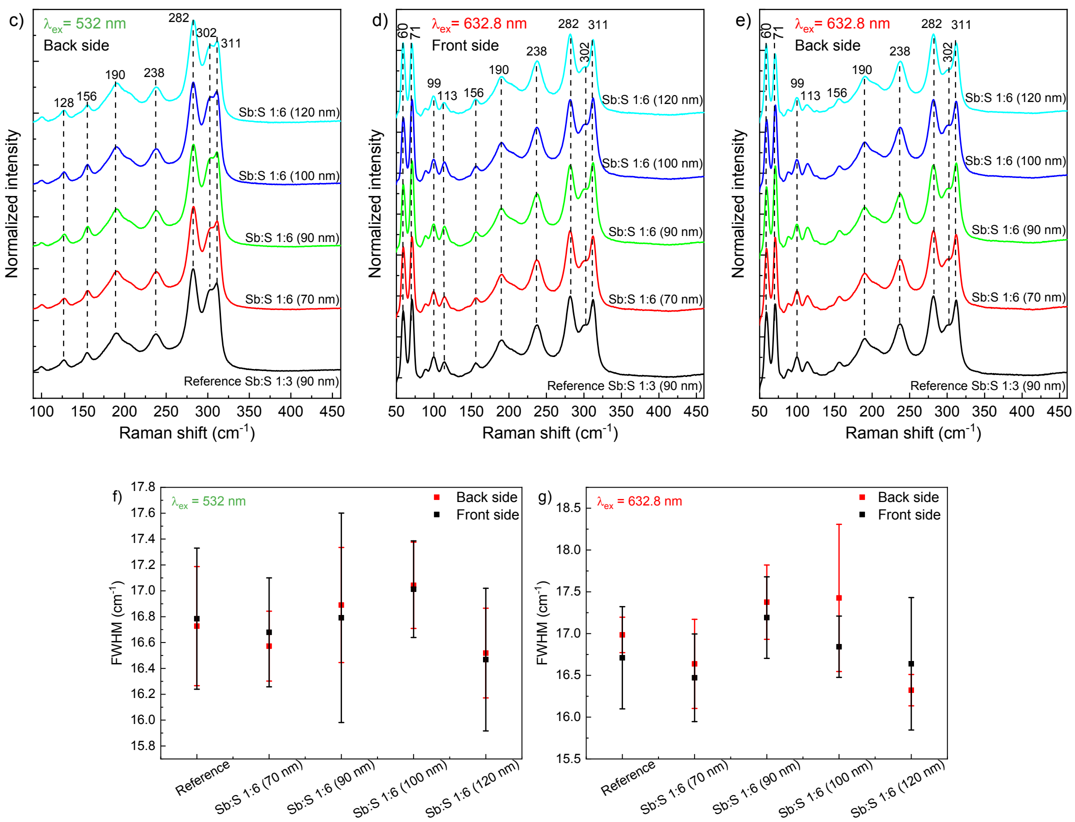

Figure S1. (a) XRD patterns (b) Raman spectra of the as-deposited reference Sb:S 1:3 (90 nm) and the Sb:S 1:6 films with varying film thicknesses. (c-e) Raman spectra of the annealed (6 min in N_2 at 270 ˚C) thin films measured under different excitation conditions. (f,g) Calculated values of full width at the half maximum of main Raman peak at 282 cm^{-1} for the annealed thin films.

XRD analysis of the as-deposited thin films (**Figure S1a**) revealed an increase in crystallinity with increasing film thickness, particularly evident in the Sb:S 1:6 films deposited for 100 and 120 cycles due to increase in deposition time.

Analysis of the Raman spectra (Figure S1 b) measured in the as-deposited thin films allows to conclude that increase of the number of deposition cycles (deposition time) and as result of the thin films’ thickness, leads to formation of partial-crystallization. This is evident from the broad band

in the range 200 – 350 cm^{-1} which is almost free of structure for the reference and two thinnest samples (70 and 90 nm), i.e. the constituent Raman peaks are too broad to be resolved, while some peak-like structure starts to appear on top of a broad band for the thicker samples (100 and 120 nm).

When measuring the Raman spectra of Sb_2S_3 under the 632.8 nm laser some additional peaks at 60, 71 and 89 cm^{-1} can be observed, this is related to the measurement setup.

Due the close to resonance measurement conditions obtained with this laser, some Raman peaks appear much intense and better resolved than in the case of the 532 nm laser, which can be observed by comparing the spectra obtained under both lasers in the region of 80 – 130 cm^{-1}.

In addition, based on the FWHM of the peak at 282 cm^{-1} calculated from both 533 and 632.8 nm Raman spectra, changes in the crystalline quality between samples are in the same range as the in-sample changes, denoting no influence of the cycle number to the crystalline quality of the annealed thin films.

Table S3. Crystallite size based on (200), (011) and (211) planes of Sb:S 1:3 reference (90 nm) and Sb:S 1:6 samples with varying film thicknesses annealed at 270 ˚C in N_2 for 6mim.

**Crystallite size (nm)** / **Sb:S (thickness)**	**(200)**	**(011)**	**(111)**	**(211)**
1:3(90nm) Reference	35 ± 5	28.1 ± 2	26 ± 3	43 ± 3
1:6 (70 nm)	24 ± 4	27 ± 6	24 ± 3	36 ± 5

1:6 (90 nm)	12 ± 5	28 ± 3	44 ± 2	35 ± 4
1:6 (100 nm)	26 ± 3	34 ± 4	33 ± 5	43 ± 2
1:6 (120 nm)	39 ± 3	26 ± 3	36 ± 4	39 ± 3

The Harris formula (Equation 1) was used to compute the texture coefficient (TC), which was used to quantitatively evaluate the degree of crystallographic orientation preference (eq S1):[1]

$$TC\ (hkl) = \frac{I(hkl)}{I_0(hkl)}\left[\frac{1}{N}\sum_{i=1}^{N}\frac{I(h_ik_il_i)}{I_0(h_ik_il_i)}\right]^{-1} \quad \text{(S1)}$$

Where N is the number of diffraction peaks, I(hkl) and I_0(hkl) are the integrated intensity ratios of the thin films and randomly orientated Sb_2S_3 powder, respectively, for a particular peak, and TC(hkl) is the texture coefficient of the plane defined by Miller indices (hkl)

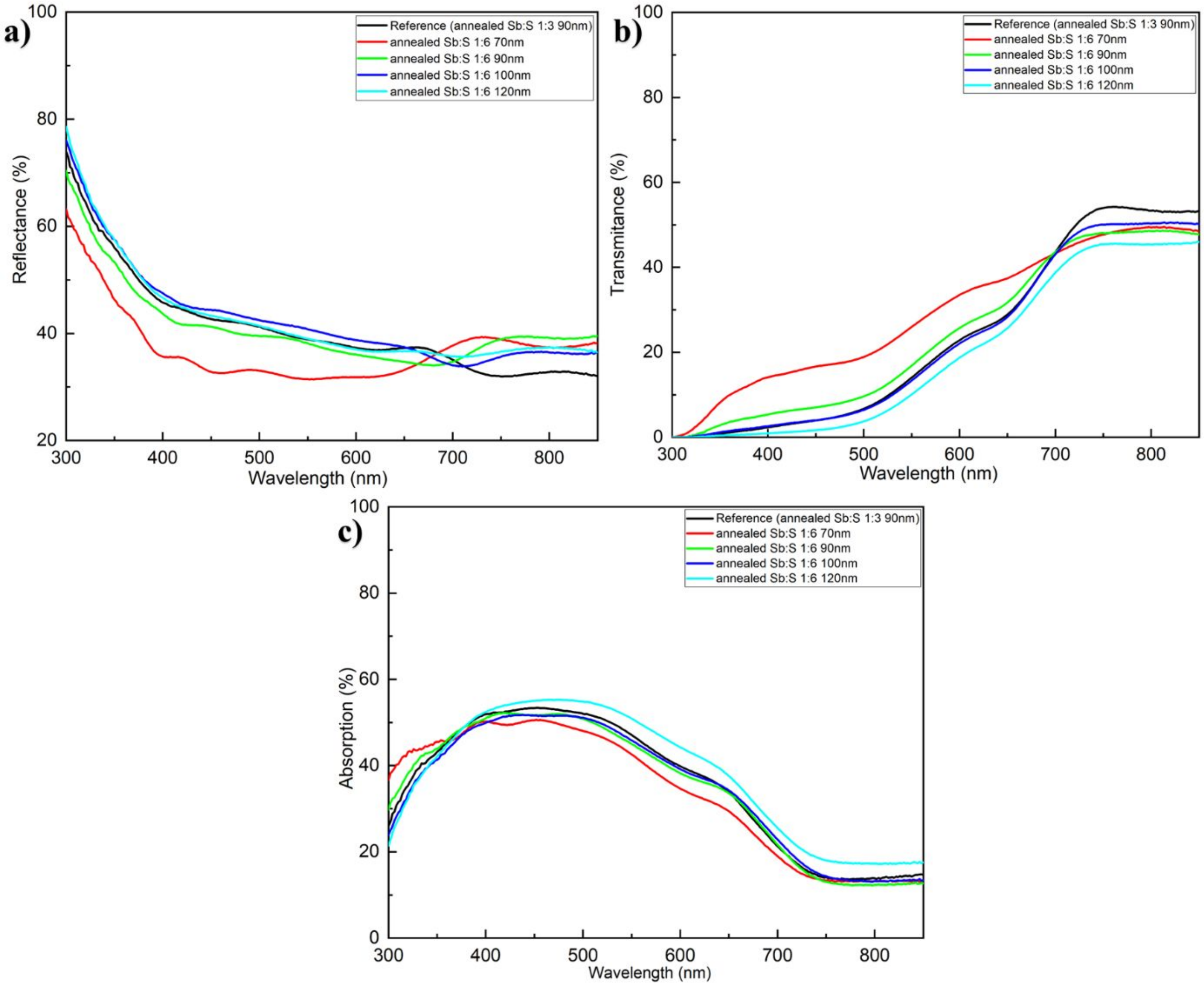

Figure S2. (a) Reflectance, (b) transmittance and (c) absorption spectra, of reference Sb:S 1:3 (90 nm) and Sb:S 1:6 (70 nm, 12 min) samples with varying layer thickness annealed at 270 ˚C inn N_2 for 6 min.

The average visible transmittance (AVT) was calculated as the arithmetic average of the total transmittance of the glass/ITO/TiO2/Sb2S3 stack in the 380-740 nm wavelength range by using Equation S2:[2]

$$AVT\ (\%) = \frac{\int_{380}^{740} T(\lambda)\, d(\lambda)}{740-380}, \qquad \text{(S2)}$$

where λ is the wavelength, and T(λ) is the transmittance at λ.

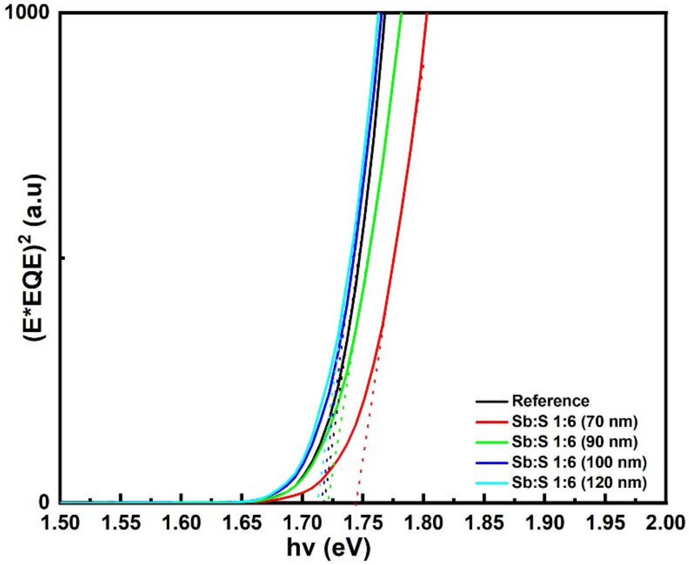

Figure S3. Optical band gaps obtain from $(E*EQE)^2$ versus *hv* for the Sb:S 1:3 reference and Sb:S 1:6 samples with varying thickness annealed at 270 ˚C in N_2 for 6 min.

Table S4. Crystallite size based on (200), (011), (111) and (211) planes of reference Sb:S 1:3 (90 nm), and 1:6 (70 nm) samples with varying annealing time at 270 ˚C in N_2.

Crystallite size (nm) / Sb:S (Annealing time)	(200)	(011)	(111)	(211)

Reference	35 ± 5	28 ± 2	26 ± 3	43 ± 3
1:6 (6 min)	24 ± 4	27 ± 6	24 ± 3	36.0 ± 5
1:6 (12 min)	--	31 ± 2	24 ± 2	44 ± 3
1:6 (18 min)	40 ± 3	18 ± 3	29 ± 3	49 ± 4

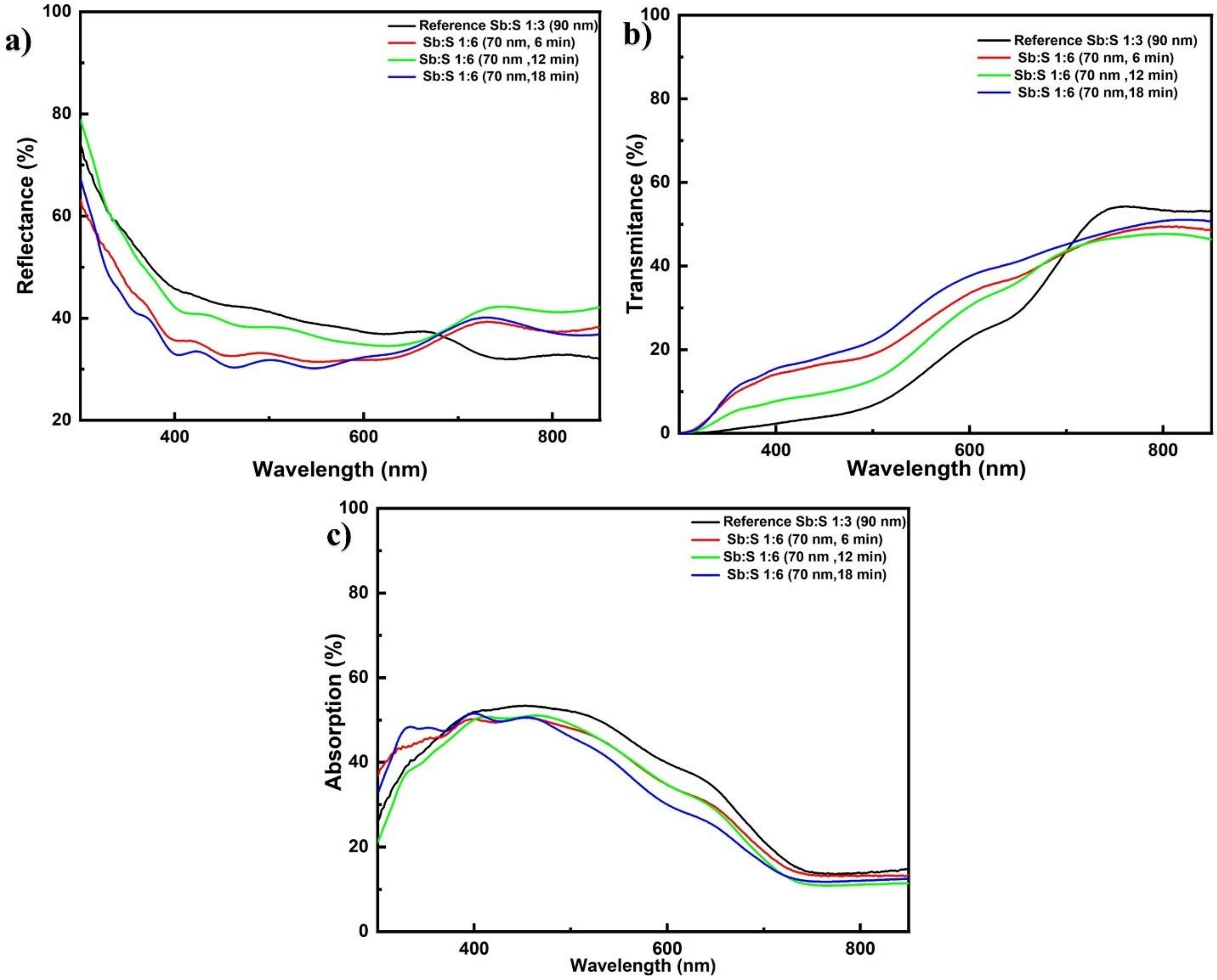

Figure S4. (a) Reflectance, (b) transmittance and (c) absorption spectra of reference Sb:S 1:3 (90 nm) and 1:6 (70 nm, 12 min) samples with varying annealing time at 270 ˚C in N_2.

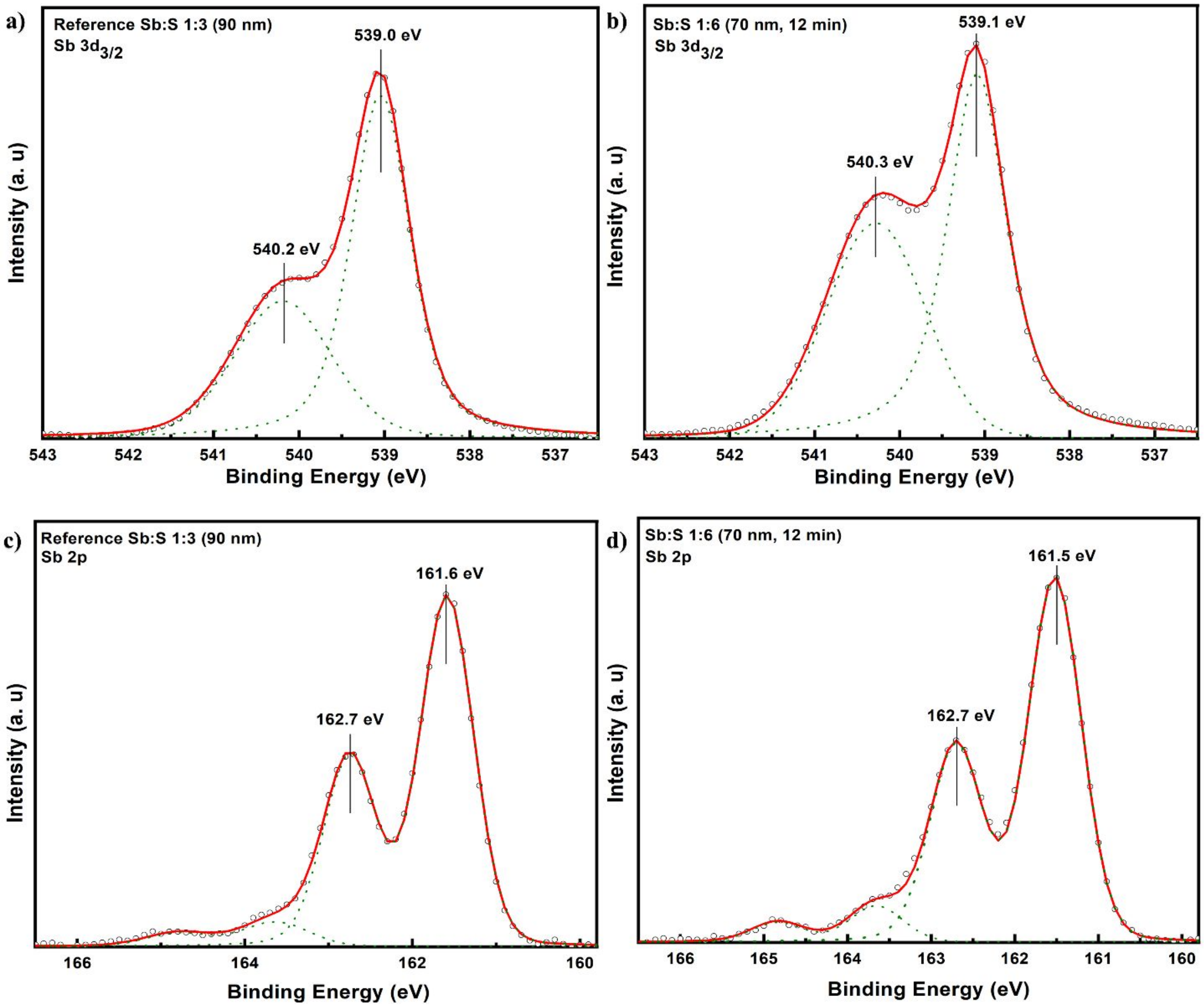

Figure S5. High resolution Sb $3d_{3/2}$ and S 2p X-ray photoelectron spectroscopy (XPS) spectra region of the surface of the (a, c) reference Sb:S 1:3 (90 nm) and the (b, d) Sb:S 1:6 (70 nm, 12 min) samples.

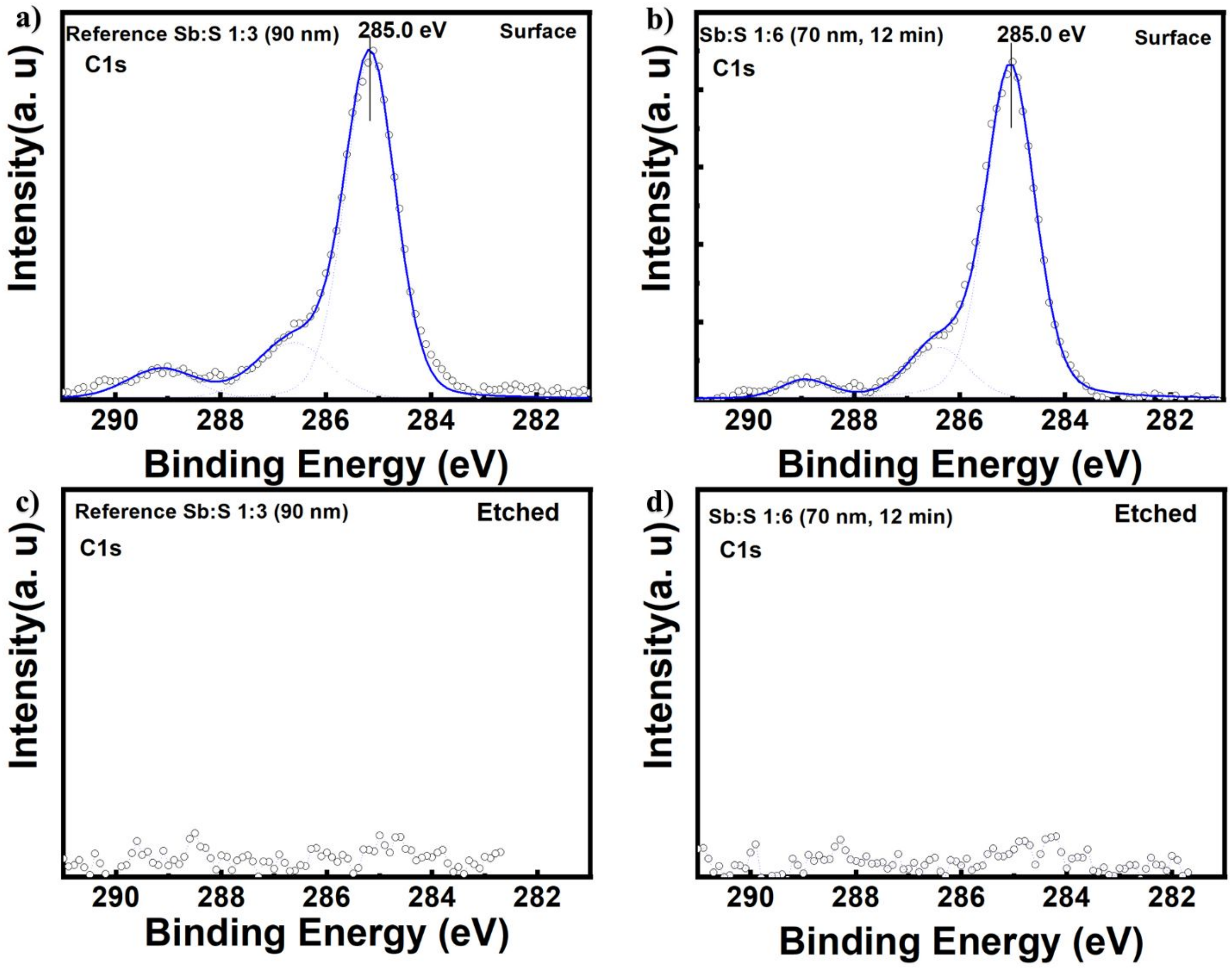

Figure S6. High resolution C 1s X-ray photoelectron spectroscopy (XPS) spectra of the (a,b) surface and (c,d) etched regions of the reference Sb:S 1:3 (90 nm) and the Sb:S 1:6 (70 nm, 12 min) samples.

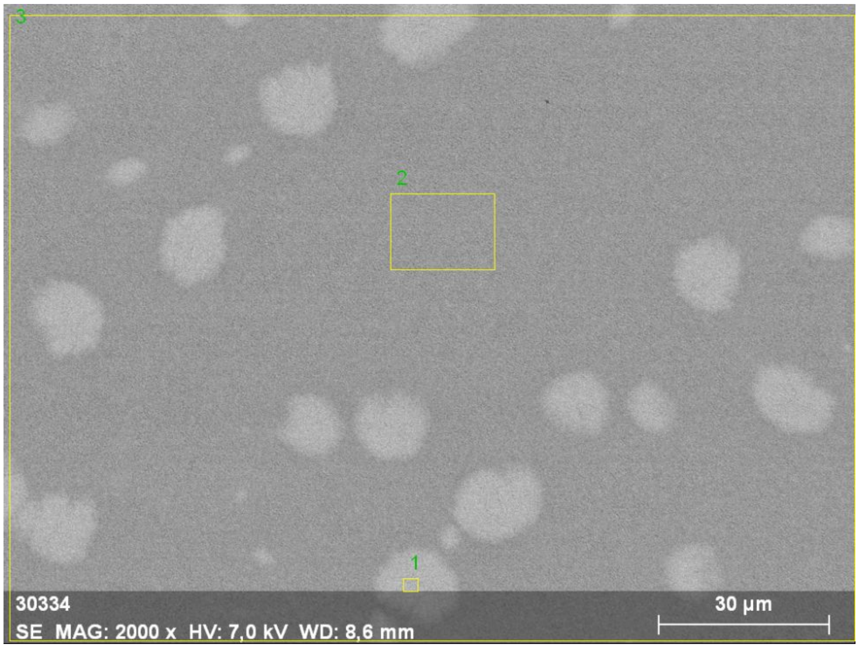

Figure S7. Scanning electron microscopy (SEM) image of the top surface of the reference Sb:S 1:3 (90 nm) film, the area of which was subsequently analyzed using energy-dispersive X-ray spectroscopy (EDS).

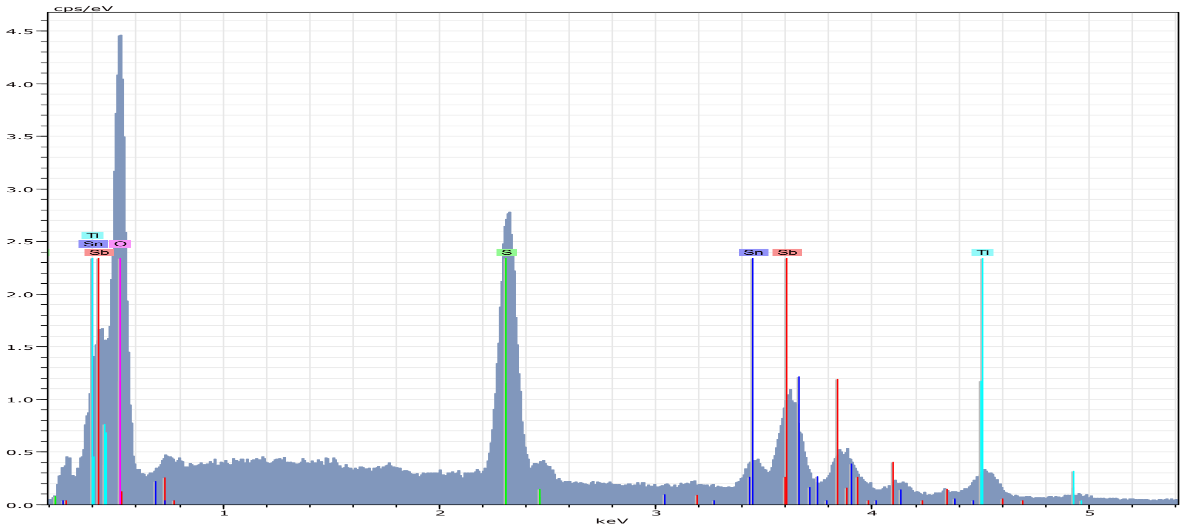

Figure S8. Energy dispersive X-ray spectroscopy (EDS) spectrum with elemental peak assignments for the reference Sb:S 1:3 (90 nm) sample obtained at a beam voltage of 7 kV.

Table S5. Elemental composition of the reference Sb:S 1:3 (90 nm) sample determined by energy-dispersive X-ray spectroscopy (EDS), showing the elements present and their corresponding atomic concentrations (%).

**Element**	**Series**	**Concentration (at.%)**	**Error (*%)**
Antimony	L-series	14.25	1.9
Sulfur	K-series	17.44	0.5
Tin	L-series	4.36	1.2
Titanium	K-series	14.08	1.0
Oxygen	K-series	49.87	26.4

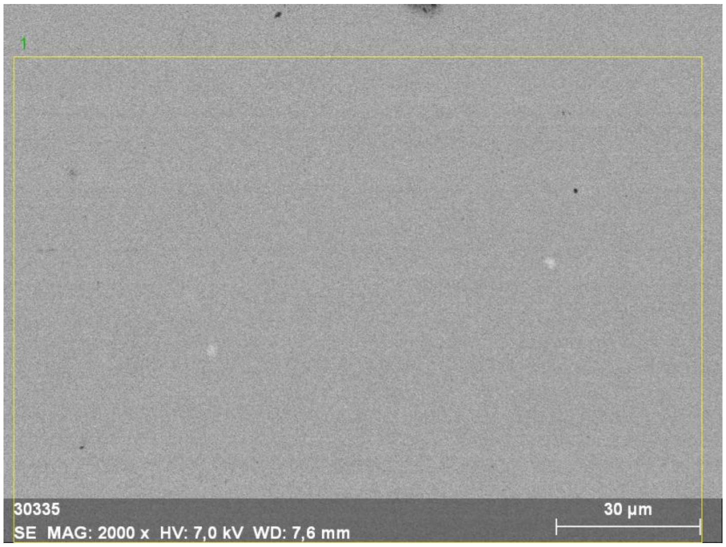

Figure S9. Scanning electron microscopy (SEM) image of the top surface of the Sb:S 1:6 (70 nm, 12 min) film, the area of which was subsequently analyzed using energy-dispersive X-ray spectroscopy (EDS).

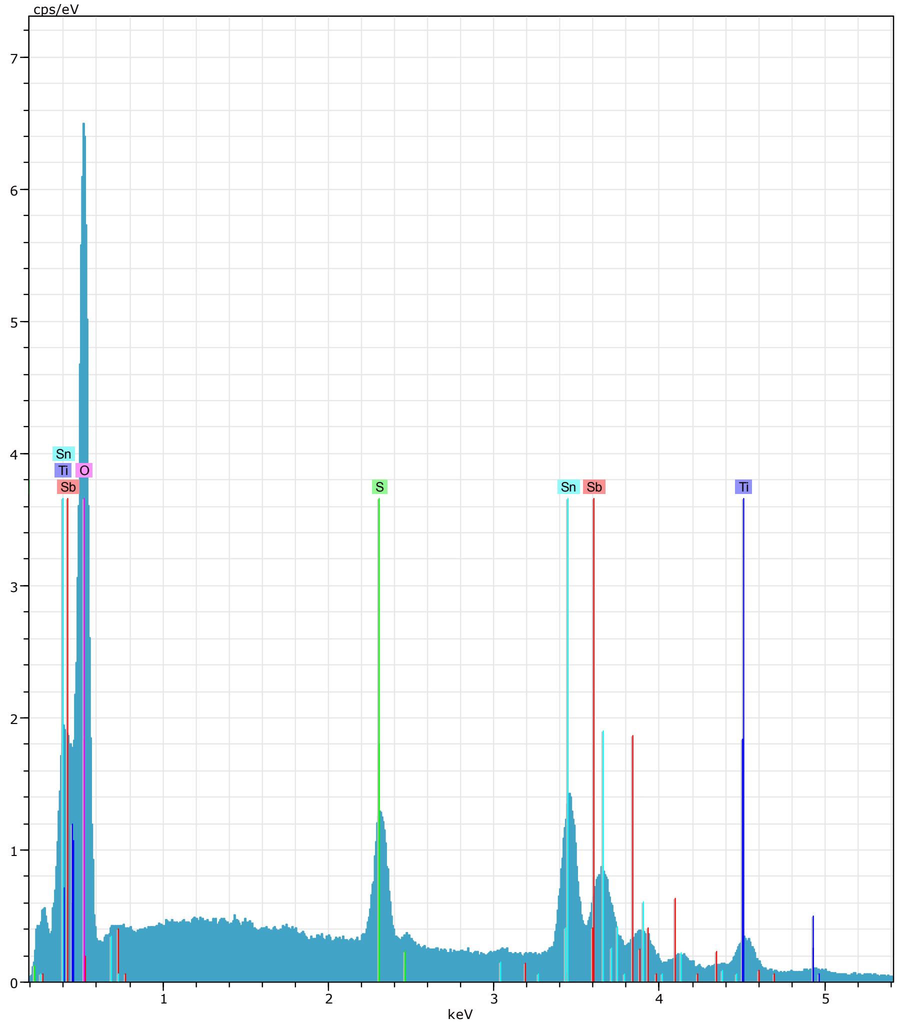

Figure S10. Energy dispersive X-ray spectroscopy (EDS) spectrum with elemental peak assignments for the Sb:S 1:6 (70 nm, 12 min) sample obtained at a beam voltage of 7 kV.

Table S6. Elemental composition of the Sb:S 1:6 (70 nm, 12 min) sample determined by energy-dispersive X-ray spectroscopy (EDS), showing the elements present and their corresponding atomic concentrations (%).

Element	Series	Concentration (at. %)	Error (%)
Antimony	L-series	4.17	0.7
Sulfur	K-series	5.95	0.2
Tin	L-series	10.06	0.7
Titanium	K-series	14.08	2.3
Oxygen	K-series	65.74	27.4

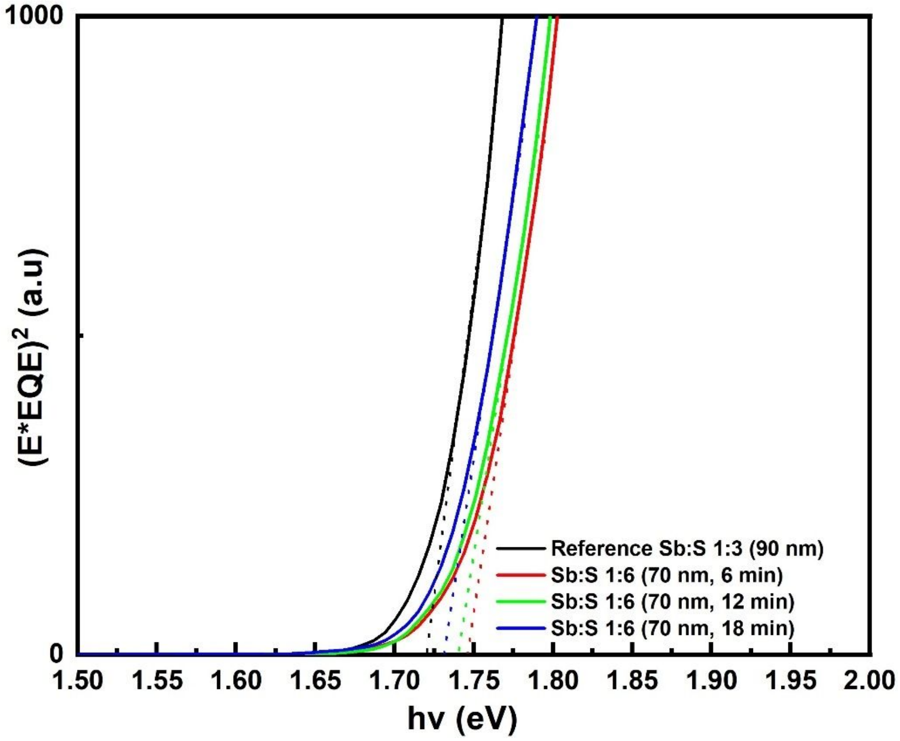

Figure S11. Optical band gap obtain from *(E*EQE)*^{2} versus *hv* for the reference Sb:S 1:3 (90 nm) and the Sb:S 1:6 (70 nm) film with varying annealing time.

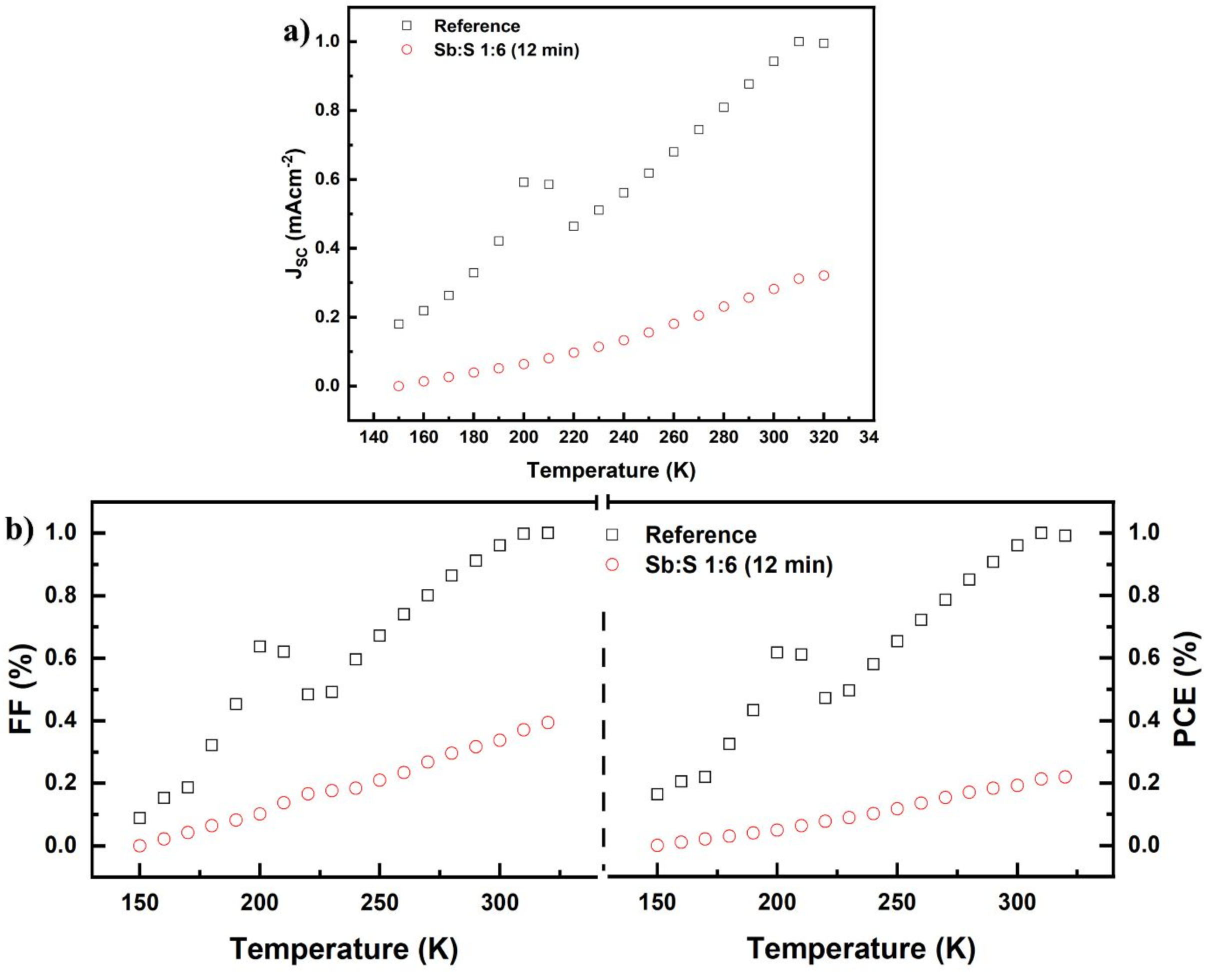

Figure S12. Temperature dependence of (a) J_{SC} and (b) *FF* and *PCE* of the reference Sb:S 1:3 (90 nm) and the Sb_2S_3 1:6 (70 nm, 12 min) solar cells.

References

(1) Spalatu, N.; Krautmann, R.; Katerski, A.; Karber, E.; Josepson, R.; Hiie, J.; Acik, I. O.; Krunks, M. Screening and Optimization of Processing Temperature for Sb2Se3 Thin Film Growth Protocol: Interrelation between Grain Structure, Interface Intermixing and Solar Cell Performance. *Sol. Energy Mater. Sol. Cells* **2021**, *225*, 111045. https://doi.org/10.1016/j.solmat.2021.111045.

(2) Eensalu, J. S.; Katerski, A.; Kärber, E.; Weinhardt, L.; Blum, M.; Heske, C.; Yang, W.; Acik, I. O.; Krunks, M. Semitransparent Sb2S3 Thin Film Solar Cells by Ultrasonic Spray Pyrolysis for Use in Solar Windows. *Beilstein J. Nanotechnol.* **2019**, *10* (1), 2396–2409.
